# pH-Mediated Solution-Phase Proton Transfer Drives Enhanced Electrochemical Hydrogenation of Phenol in Alkaline Electrolyte

**DOI:** 10.1021/acscatal.4c04874

**Published:** 2024-11-01

**Authors:** Brianna Markunas, Taber Yim, Joshua Snyder

**Affiliations:** Chemical and Biological Engineering, Drexel University, Philadelphia, Pennsylvania 19104, United States

**Keywords:** electrochemical hydrogenation, phenol, biomass conversion, platinum, rhodium

## Abstract

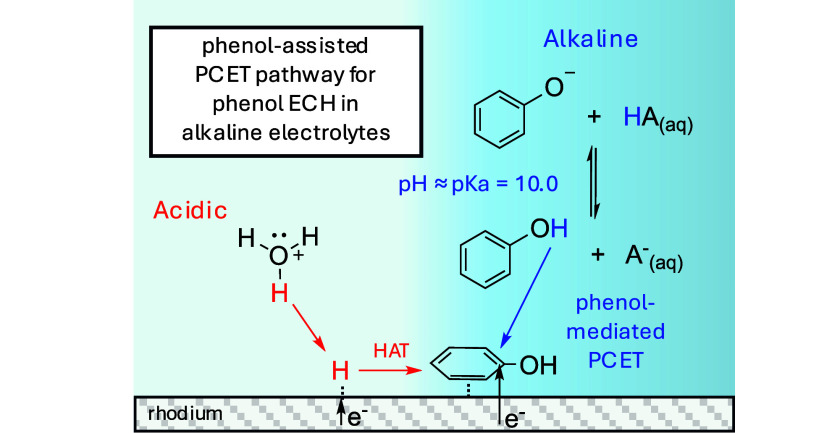

The faradaic efficiency (FE) of the electrochemical hydrogenation (ECH) of phenol and other biomass-derived model compounds could potentially be improved by operating in alkaline electrolytes, where the parasitic hydrogen evolution reaction rate is significantly slower due to a higher Volmer step barrier. However, this approach is potentially limited by the impact of the higher barrier for adsorbed hydrogen (H_ad_) formation, as hydrocarbon ECH is expected to be limited by a hydrogen atom transfer, progressing through a Langmuir–Hinshelwood-type (LH) mechanism. In this work, we show that there are contrasting pH trends for phenol ECH between Pt and Rh, two common catalysts for ECH reactions. Phenol ECH FE and rate on Pt is highest in acidic electrolytes of pH ≤ 5, while activity on Rh is highest near pH 9–10. While our kinetic analysis supports a LH mechanism for Pt at all pH, phenol ECH on Rh shifts from a LH mechanism at low pH to being limited by a direct proton-coupled electron transfer (Eley–Rideal-type mechanism) in which surface adsorbed phenol is hydrogenated by solution-phase H-transfer. We show that the peak activity on Rh at pH 9–10 is due to the proximity of the pH to the p*K*_a_ of phenol (p*K*_a_ = 10.0). The reversibility of protonation/deprotonation of phenol when electrolyte pH matches its p*K*_a_ helps to mediate H-transfer from solution to adsorbed phenol. We also discuss the role of buffer species in mitigating the local pH change and as a H-donor in phenol ECH on Rh at alkaline pH.

## Introduction

The chemical and petroleum industries account for a large fraction of the energy consumption in the industrial manufacturing sector.^1^ A reduction in energy consumption in this sector, driven by improvements in the process efficiency, will have significant impacts on future global energy needs as well as environmentally deleterious carbon output. The requisite high temperature and pressure conditions for traditional heterogeneously catalyzed chemical conversions, of which thermal chemical hydrogenation (TCH) is a key reactive process, limits the energy efficiency of petroleum and biomass feedstock conversion to value-added fuels and chemicals.^2−4^ TCH also requires externally produced H_2_, which further contributes to the process energy intensity and carbon output. Significant reductions in the energy input as well as the carbon output can be achieved by reducing the required process temperatures, improving selectivity, and sourcing complex chemical species from renewable sources such as biomass. Electrochemical hydrogenation (ECH) is an attractive alternative where applied potential drives the hydrogenation of hydrocarbons with protons sourced from water in the aqueous electrolyte at near ambient conditions.^5−7^ Shifting away from nonrenewable petroleum-derived chemical precursors, lignocellulose is an abundant yet underutilized renewable feedstock comprised of complex phenolic compounds that can be valorized via hydrogenation.^5,8−11^ The applied potential in ECH systems introduces an additional parameter that can be tuned to control selectivity, which is crucial for biomass feedstocks that have a highly variable composition. Of the hydrocarbon components that can be derived from the breakdown of lignocellulose, phenol represents a simple model compound that has been the focus of recent ECH studies.^12−22^ Phenol and its derivatives are important commodity and precursor chemicals for many materials and industries.^23^ Upgrading of phenol through TCH or ECH yields two main products, cyclohexanone and cyclohexanol,^2,24^ which are both precursors in the production of resins, plastics, nylon-6,6, etc.^25^

Rhodium (Rh) and platinum (Pt) are common phenol ECH electrocatalysts due to their ability to adsorb both phenol and hydrogen with reasonable adsorption strengths.^18,26^ Several studies of phenol ECH on Pt and Rh indicated that it follows the same Langmuir–Hinshelwood mechanism as in TCH, in which surface-adsorbed phenol is hydrogenated by neutral surface-adsorbed hydrogen in a hydrogen atom transfer (HAT) step.^12−14^ The rate-determining step was suggested to be the first H-addition to the aromatic ring.^14^ It was shown that at high conversion rates, phenol ECH is selective toward cyclohexanol with cyclohexanone as an intermediate.^12,13,27^ Existing limitations for ECH of phenol include a direct competition with the hydrogen evolution reaction (HER) leading to a reduced faradaic efficiency (FE) [40% for Pt^14^ and 70% for Rh^13^ at −0.3 V vs the reversible hydrogen electrode (RHE) in pH 5 acetate buffer], incomplete understanding of the role of electrolyte and the properties of the electrochemical interface on reaction rates/selectivity, and reports of ECH turnover frequencies on Rh/C in a pH 5 acetate buffer that are only competitive with TCH rates at overpotentials of −0.7 V vs Ag/AgCl or higher.^13^

There are recent efforts to improve phenol ECH activity that have focused on varying the composition of bimetallic or metal/metal-oxide interfaced catalysts where the targeted design criteria is the balancing of the adsorption strength and coverage of both phenol and hydrogen.^15,17,20,28,29^ FEs of nearly 50% in 1 M H_2_SO_4_ on a Ni–MoO_2_ catalyst has been attributed to the enhanced adsorption of phenol over Ni alone.^15^ One group observed a maximum FE of 87% on their Pt_3_Ru_3_ alloy,^17^ while another group observed improved FE and selectivity toward cyclohexanol on both their Pt_3_Ru and Pt_3_RuSn catalysts.^20^ Similar trends have been observed on PtRh alloys, where one group found that increasing the ratio of Rh increased the FE up to 86%, which they attributed to the enhanced specific adsorption of phenol and weakened interaction between H_ads_ and the alloy.^29^ Singh et al. recently studied phenol ECH on PtCo alloys. They observed higher phenol ECH rates on a PtCo alloy due to increased phenol surface coverage resulting from a weakened hydrogen adsorption energy. The HER activity, however, was also higher due to this weaker H_ads_ and resulted in lower FE for phenol ECH.^21^ These composition-dependent performances have been attributed to the optimization of the surface by strengthening the adsorption of phenol and weakening the binding of hydrogen.^15,28,29^ Better balancing of the competitive adsorption of hydrogen and phenol leads to greater phenol coverage and higher ECH rates.

Currently, comparative assessment of reaction rates presented on different catalyst materials from different groups is challenging at best. As with many other electrochemical reactions/systems, the lack of standardizations in terms of material preparation, testing, analysis, quantification, and normalization results in limited meaningfulness to any comparisons. For electro–organic reactions, this can be exacerbated by the complexity of electrolyte additives (i.e., buffering species), variance of applied potentials used, electrode morphology, and the potential for dramatic pH changes within the electrolyte during extended electrolysis. Additionally, the difficulty in accurately determining the true surface area of electrocatalysts injects large degrees of error into any turnover number/rate calculations. Outside of these experimental challenges, much of the data analysis and performance metric determination is predicated on HAT in a Langmuir–Hinshelwood type mechanism as being rate limiting.^13,14^ Recently, Sun et al. suggested that phenol ECH on their PtRu catalyst follows an Eley–Rideal (ER) mechanism in which adsorbed phenol is hydrogenated through a proton-coupled electron transfer (PCET) by protons from the electrolyte.^17^ This highlights an existing knowledge gap where the direct impact of electrochemical system parameters on ECH mechanisms, rates, and selectivity is currently incompletely understood.

Beyond some work on the structural sensitivity of phenol ECH, through variance of particle size^30,31^ and employing adsorption isotherms on polycrystalline Pt,^32^ there is limited work on the role of other aspects of the electrochemical reaction environment. Song et al. studied the effect of different electrolytes at pH 5, finding that acetic acid/acetate showed the highest activity for phenol ECH on Rh and Pt, followed by phosphoric acid/phosphate and sulfuric acid/sulfate.^12^ Limited analysis of the effect of electrolyte pH, through measuring phenol ECH at pH 3, 5, and 10, showed that on Pt, the phenol ECH rate and FE peaked at pH 5 and decreased at pH 10. In contrast, they found that the highest phenol ECH activity on Rh was at pH 10, with a FE of 95%. They hypothesized that this could be due to pH-dependent adsorption of phenol, pointing to the dissociation of phenol to phenoxide at pH 10.^12^ Despite this result, the true source of activity enhancement on Rh in an alkaline electrolyte remains unknown. This is partly due to the fact that much of the published work on phenol ECH has been performed within the pH range 1–5.^12−20^ This is not surprising if we assume that ECH of phenol is governed by an HAT-mediated LH mechanism.^13,14^ However, the role of pH in phenol ECH is likely much more complex due to the presence of the electric fields from applied potential, whose strength varies with pH, and its role in affecting both solvent and reactant molecule interaction with the surface.^33−35^ Electrolyte pH will also affect the rate of surface-adsorbed H formation, with barriers for Volmer-type reactions increasing with increasing pH.^33,36,37^ Additionally, for hydrocarbon reactants with functional groups, the p*K*_a_ of those functional groups can be within the range of electrolyte pH. This is true for phenol, which has a p*K*_a_ of 10.0. This means that at electrolyte pHs approaching and surpassing 10.0, phenoxide begins to become the dominant form of the reactant, which would be expected to have a considerable impact on reaction kinetics.

In this paper, we explore the role of electrolyte pH on two common phenol ECH catalysts, Pt and Rh. It can be reasonably expected that as electrolyte pH is increased, HER rates will decrease as the Volmer step reactant shifts from H_3_O^+^ to H_2_O.^38^ The desired effect in running phenol ECH at alkaline pH is improved selectivity over the parasitic HER, resulting in an improved FE for phenol ECH compared to that in acidic pH electrolytes. Here, we show that there are contrasting trends in pH-dependent ECH rates and FE for Pt and Rh. ECH is found to be the most efficient on Pt in acidic electrolytes, while the peak performance for Rh is found at pH 10, near the p*K*_a_ of phenol. The peak performance on Rh outpaces that on Pt with both a higher FE and phenol ECH partial current density (PCD). We show that this pH-dependent behavior is a convolution of the rate at which adsorbed hydrogen is formed on the surface, differences in potential-dependent phenol/hydrogen coverage on Pt and Rh, competition with HER, and pH-driven changes to the balance between HAT- and -mediated ECH mechanisms. With this insight, we show that the unique properties of Rh and the p*K*_a_ of phenol can be exploited to yield elevated ECH rates with an enhanced FE.

## Methods

### Cell/Electrolyte Preparation and Electrochemical Testing

A single chamber FEP cell with a Pt counter electrode and a double junction-chambered Ag/AgCl reference electrode (Basi, 3 M KCl) was used for cyclic voltammetry (CV) measurements. The cell was filled with 40 mL of electrolyte, and the desired concentration of phenol was added directly and stirred prior to the CV measurement. CVs are reported following the IUPAC convention. A custom-made H-Cell (Figure S1) was used for electrolysis measurements. It consisted of a Ag/AgCl reference inside a double junction and a Pt or Rh working electrode in the catholyte chamber. A Pt counter was used in the anolyte chamber and a Nafion N117 membrane or a Versogen PiperION anion exchange membrane (20 μm, Fuel Cell Store) separating the two chambers. Each chamber was filled with 15 mL of electrolyte. The catholyte contained 15 mM phenol for all electrolysis experiments, unless stated otherwise, and was stirred with a magnetic stir bar at 700 rpm. All electrochemical cells for both CV and electrolysis measurements were initially soaked in a concentrated H_2_SO_4_ and HNO_3_ (1:1) solution for 10 h followed by three rounds of rinsing and boiling in deionized (DI) water (Milli-Q). The cells were rinsed thoroughly and boiled with DI water between all tests. All electrochemical cells were purged with argon 10 min before and during measurements. The Ag/AgCl reference electrodes were calibrated to the RHE and all potentials reported in this work are reported against the RHE. Buffered electrolytes were used across the pH range, with the exception of 0.1 M HClO_4_ or 0.1 M H_2_SO_4_ for pH 1 and 0.1 M KOH for pH 13. Theoretical titration curves were calculated for each of the buffers and used to perform a backward titration of 0.6 M buffer acid with the calculated amount of potassium hydroxide (KOH) (Sigma-Aldrich, semiconductor grade, 99.99% trace metals basis) to achieve the targeted pH and 0.1 M ionic strength. A 0.1 M KClO_4_ supporting electrolyte was used when the 0.1M ionic strength could not be achieved simultaneously with the desired buffer concentration. Additional details about the buffer preparation can be found in the Supporting Information. The buffer species used were phosphoric acid (Sigma-Aldrich, 85 wt % in H_2_O, 99.99% trace metals basis), acetic acid (Sigma-Aldrich, ≥99.99% trace metals basis), boric acid (Beantown Chemical, 99.99% trace metals basis), or potassium bicarbonate (Sigma-Aldrich, ≥99.95% trace metals basis). Table 1 summarizes the buffers used at each pH in this work unless otherwise stated. The buffer systems at each electrolyte pH were chosen to be close to the p*K*_a_ of the buffer acid to minimize variations in buffering capacity, which depends on the proximity of pH to the buffer p*K*_a_. The pH change measured before and immediately after electrolysis was no more than +0.25 pH units in each buffer.

**Table 1:** Buffer Systems Used at Each pH for Phenol ECH

pH	buffer	buffer ions present	p*K*_a_
3	phosphoric acid/phosphate	H_3_PO_4_, H_2_PO_4_^–^	2.16
5	acetic acid/acetate	CH_3_COOH, CH_3_COO^–^	4.76
9	boric acid/borate	H_3_BO_3_, H_2_BO_3_^–^	9.2
10	potassium bicarbonate/carbonate	KHCO_3_, CO_3_^2–^	10.3
11	potassium bicarbonate/carbonate	KHCO_3_, CO_3_^2–^	10.3
12	phosphate	HPO_4_^2–^, PO_4_^3-^	12.35

All electrochemical measurements were performed by using an Autolab PGSTAT302N potentiostat with 85% *iR* compensation applied, as determined by electrochemical impedance spectroscopy measurements. The electrolytes, cell configurations, and electrodes to be used are discussed above and below. CVs and extended electrolysis were used to characterize the electrodes and perform electrochemical reaction analysis.

### Electrode Preparation

**Figure 1:**
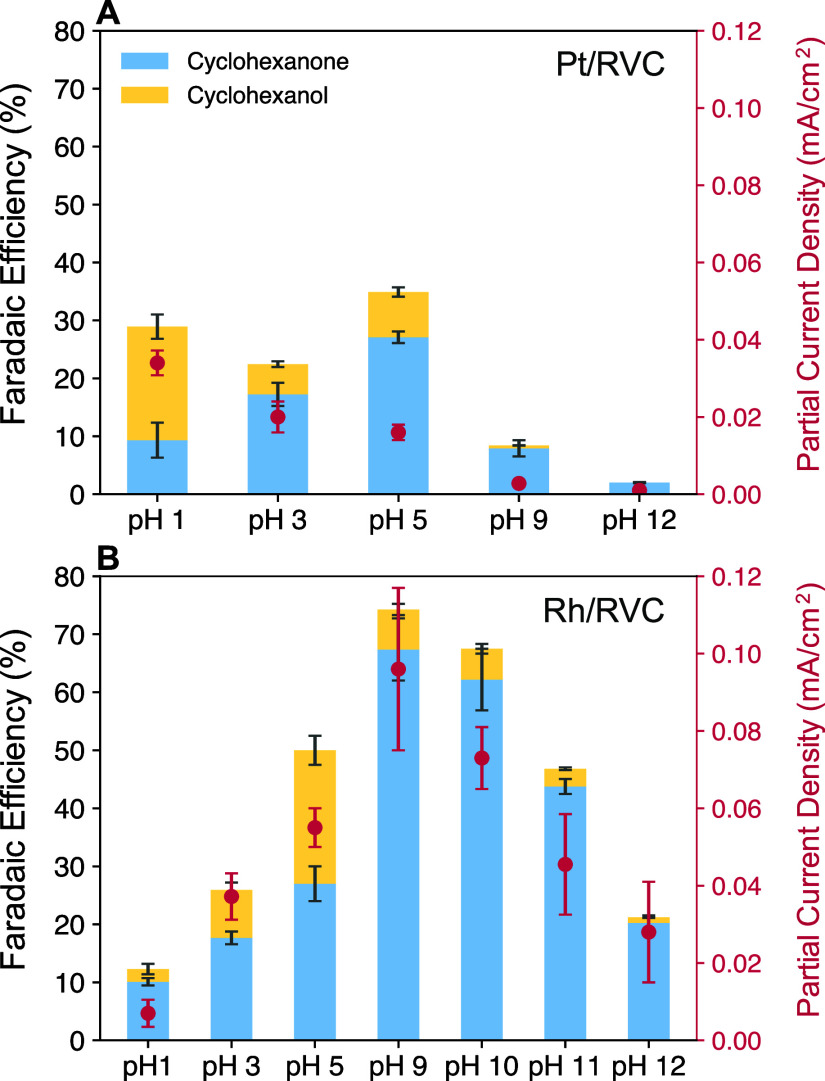
FE (%) and PCD (mA/cm^2^) for phenol ECH on (A) Pt/RVC and (B) Rh/RVC after extended electrolysis at −0.2 V vs RHE for 1 h with 15 mM phenol in the catholyte. The error bars represent standard deviation of at least 3 replicates. See Figure S13 for the corresponding carbon balance.

A Pt wire (Surepure Chemetals, 1.0 mm diameter, 99.999%) or Rh wire (Millipore Sigma, 1.0 mm diameter, 99.9%) was used for CVs. The wires were dipped in concentrated HNO_3_, rinsed, and sonicated in DI water before each electrochemical measurement. The wires were electrochemically cycled from 0.05 to 1.1 V vs RHE at a scan rate of 250 mV/s for 50 cycles, or until a steady-state response was reached, prior to taking CVs at 50 mV/s. The electrochemically active surface area (ECSA) was estimated by hydrogen underpotential deposition (H_UPD_) charge, using the hydrogen desorption peak on Rh because the hydrogen adsorption peak overlaps with Rh_2_O_3_ reduction.^26^ For electrolysis experiments (Figure 1), the working electrode was either Pt or Rh electrodeposited onto a 0.5 in. × 0.5 in. × 0.5 in. cube of reticulated vitreous carbon (RVC) mesh (Duocel, 60 pores per inch). RVC was chosen as the catalyst support due to its high surface area, a highly uniform pore size and distribution, and high conductivity. The RVC mesh was attached to a graphite rod (Sigma-Aldrich, 99.95% trace metals basis) and then rinsed and sonicated in DI water. The bare RVC WE was inserted into a single chamber FEP cell containing 5 mM H_2_PtCl_6_ × 6H_2_O or 5 mM RhCl_3_*x*H_2_O in a 0.1 M NaCl solution. Electrodeposition of Pt onto the RVC was achieved by pulsing the potential to −0.3 V vs RHE for 20 constant charge intervals. The same procedure was followed for Rh but pulsed to −0.2 V vs RHE. Following electrodeposition, the RVC mesh was visibly and uniformly coated with metallic Pt or Rh (Figure S4). The electrodes were then rinsed thoroughly and sonicated in DI water and then transferred to another FEP cell containing fresh 0.1 M HClO_4_, where a CV was taken and the ECSA was calculated by H_UPD_. Details of the ECSA calculation and the average ECSA of the prepared electrodes are provided in the Supporting Information (Table S1). Figures S6 and S7 show scanning electron microscopy images with colorized elemental mapping using energy-dispersive spectroscopy of the Pt/RVC and Rh/RVC electrodes after preparation, displaying a uniform coverage of the metal over the RVC. Figures S8 and S9 show the sizes of metal aggregates following Pt and Rh electrodeposition on RVC. The electrodes were rinsed with DI water again following CV measurements. For the electrolysis experiments used to obtain the data in Figure 3, the WE was a Pt or Rh wire prepared in the same manner as that described for CV measurements.

### Product Analysis

Same-day product analysis of the phenol ECH electrolysis samples was performed using an Agilent 5977B-GC/-MSD with an HP-5 ms ultra inert capillary column. A liquid–liquid extraction was performed by using 3 mL of the aqueous electrolyte samples and an equal volume of dichloromethane. The organic phase was dried with Na_2_SO_4_. At low conversions where the cyclohexanol yield was low, the sample was silylated with excess *N*,*O*-Bis(trimethylsilyl)trifluoroacetamide (BSTFA) in the organic phase to increase its sensitivity in the GC–MS. A calibration curve was created by preparing standards undergoing the same extraction and silylation workup. The phenol concentration after electrolysis was also quantified. No phenol or phenol hydrogenation/oxidation products as a result of crossover into the anode compartment were detected by GC–MS.

## Results and Discussion

ECH electrolysis of phenol is performed on Pt/RVC under constant potential at −0.2 V vs RHE for 1 h. At −0.2 V vs RHE, we expect a sufficiently high turnover of phenol without excessive HER. Figures S10 and S11 show the suppression of H_UPD_ on Pt and Rh in the presence of phenol in each buffer/pH used for electrolysis, indicative of phenol adsorption on the metals across the pH range. The FE and PCD are calculated using the charge passed and GC–MS product analysis, with the remaining balance of the charge attributed to the HER. The two main products of phenol hydrogenation are cyclohexanone and cyclohexanol. No other products, including deoxygenation to cyclohexane, are observed in this work. The FE and PCD for hydrogenation products on Pt are shown in Figure 1A. The results show that on Pt, the FE peaks at pH 5 and PCD peaks at pH 1, both then significantly decrease with increasing pH. The phenol conversion rate at each pH (Figure S12A) follows the same trend as the PCD. This is in agreement with the lower rates for phenol ECH on Pt in pH 10 acetate buffer^12^ and the negligible phenol ECH rates on PtRu in 0.2 M KOH.^17^

**Figure 2:**
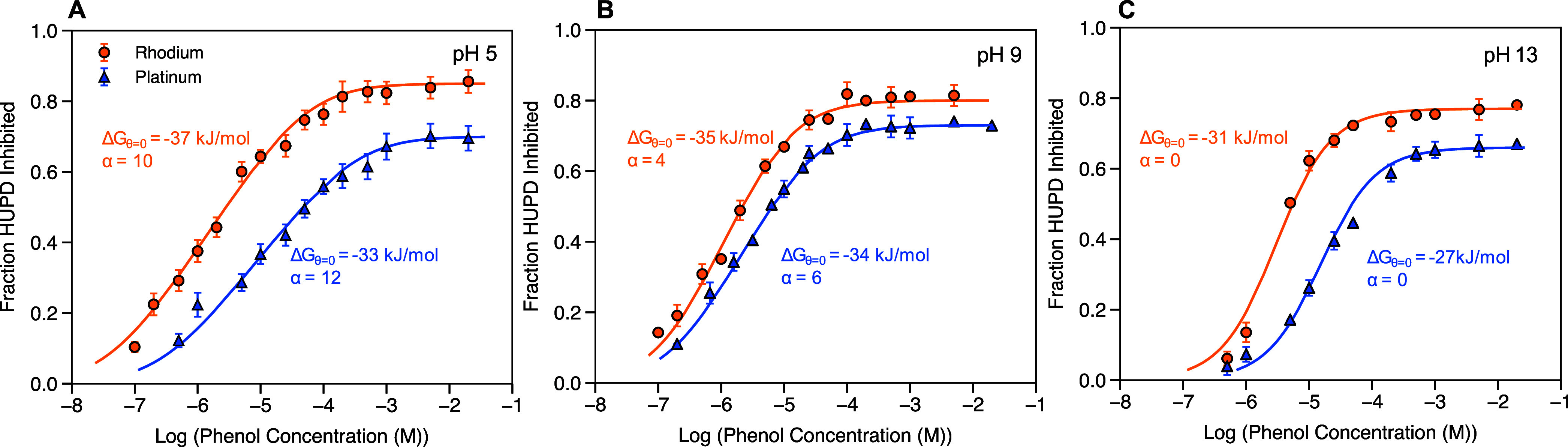
Fraction of H_UPD_ inhibited vs bulk phenol concentration on Rh wire (orange circles) and Pt wire (blue triangles) in (A) pH 5 (0.6 M acetic acid/acetate buffer), (B) pH 9 (0.6 M boric acid/borate buffer), and (C) pH 13 (0.1 M KOH). Error bars represent standard deviation of 2 replicates. Cyclic voltammograms measured in the absence and presence of vary phenol concentrations to obtain the fraction of H_UPD_ inhibited are shown in Figures S15 and S16.

It has been proposed that phenol ECH on Pt follows a Langmuir–Hinshelwood (LH)-type mechanism,^14^ in which surface-adsorbed phenol is hydrogenated by surface-adsorbed hydrogen (H_ads_) in a HAT step. While the coverage of H_ads_ is not a function of pH in the absence of phenol, the barrier for its electrochemical formation through the Volmer step increases with increasing pH as the source of H_ads_ shifts from H_3_O^+^ to H_2_O.^33,37,38^ This is the likely source of the pH dependence of HER electrocatalysis on Pt.^37,39−41^ It is this increased barrier to HER that we wish to take advantage of to improve the ECH FE for phenol. However, H_ads_ is also a reactant for phenol ECH if proceeding through a LH mechanism. On Pt, the observed result is a reduction in FE and PCD of ECH, and even overall measured current density (Figure S14), with increasing pH. Phenol ECH on Pt is already likely limited by the coverage of H_ads_ as adsorbed phenol coverages at a broad range of solution concentrations are found to be high.^26^ This limiting behavior has been seen in other hydrogenation systems, such as the electrochemical reduction of CO_2_ to CH_4_.^42^ Following an LH mechanism, the lower coverage of H_ads_ in the presence of phenol and the increased barrier for H_ads_ formation at higher pH values can explain the observed decrease in the PCD of phenol ECH at alkaline pH on Pt. The reduced ECH FE for Pt at alkaline pH is suggestive of a lower barrier for H–H recombination (Tafel step) than an HAT-mediated ECH of adsorbed phenol. The slight increase in FE at pH 5 could be due to the moderate inhibition of HER at pH 5 compared to pH 1 and 3, where phenol ECH rates are still appreciable, before becoming negligible at more alkaline pH. Other factors that may play a role include a pH dependence on the coverage and adsorption free energy of phenol (see Figure 2), as well as the change in the proportion of phenol and phenoxide in solution as the electrolyte pH approaches and surpasses the p*K*_a_ of phenol. This can be particularly impactful as the surface charge on the electrode is likely negative at operational potentials for ECH (potential of zero total charge for Pt is 0.3 V at pH 1 and ∼0.7 V at pH 13^43^). On Pt(111), which has been suggested to be the most active facet for phenol ECH,^26^ it has been established that a benzene ring adsorbs parallel to the surface through its π electrons.^44^ Li et al. computationally demonstrated that on Pt(111), the aromatic benzene ring of phenol adsorbs in this parallel configuration while its –OH group is tilted upward, away from the surface. They show that when phenol dissociates to phenoxide, the –O^–^ group tilts even further away, increasing the bond distance between the α carbon and the Pt surface.^45^ This dissociation-dependent shift in the adsorption geometry, exacerbated by the electrostatic repulsion induced by the negatively charged catalyst surface, is likely to increase the barrier for hydrogenation. A similar argument has been made for aromatic hydrocarbons like benzaldehyde where the weaker interaction of the benzaldehyde ring with the metal due to steric hindrance from the aldehyde group is blamed for lack of hydrogenation of the aromatic ring during benzaldehyde ECH.^18^

The ECH of phenol is performed on Rh/RVC under the same conditions as those for Pt/RVC. The FE and PCDs on Rh are shown in Figure 1B. In contrast to Pt, our results show that ECH activity peaks near pH 9 on Rh, with an average FE of 74% and an ECH PCD of 0.096 mA/cm^2^. This agrees with prior results, where phenol ECH at pH 3, 5, and 10 using acetate buffers showed that ECH activity on Rh increased with increasing electrolyte pH, surpassing the ECH activity on Pt in acidic electrolytes.^12,18^ While being previously observed, the pH dependence of phenol ECH on Rh remains poorly understood. The observed peak in ECH performance at an electrolyte pH near the p*K*_a_ of phenol on Rh but not on Pt is an interesting result, requiring further analysis.

The unique pH dependence for phenol ECH on Rh, in direct contrast to that observed on Pt, could be a consequence of the difference in the interaction between the catalyst, phenol, and electrolyte induced by the different properties of the metals. The hydrogen binding energy (HBE) is slightly stronger on Rh (−0.30 eV) than Pt (−0.27 eV).^46^ Previous works on the pH dependence of phenol TCH on Pt suggested that the decrease in the hydrogenation rate at higher pH was due to the increase in HBE^47^ with increasing pH, slowing the HAT-mediated hydrogenation of adsorbed phenol. It has now been shown, definitively, that HBE is intrinsic to the catalyst and not a function of pH.^48−51^ The small difference in HBE on Rh and Pt is unlikely enough to explain the observed difference in pH-dependent ECH behavior. Phenol adsorption isotherms have been previously employed by Akinola et al. to gain insights into the adsorption free energy and coverage of phenol on Pt wire and Rh wire in pH 5 acetate buffer.^26^ They fit phenol adsorption isotherms to a two-site Temkin adsorption model and found that the free energies for phenol adsorption on Pt and Rh are similar.^26^ In Figure 2, we show adsorption isotherm plots for phenol on Pt and Rh at pH 5, 9, and 13, assuming that the observed reduction in H_UPD_ charge with increasing phenol concentration is proportional to phenol coverage.^26^ The plots of fraction H_UPD_ inhibited versus phenol concentration ranging from 0 to 20 mM are fitted with a Temkin adsorption isotherm
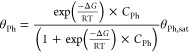
1
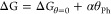
2where θ_Ph_ is phenol coverage, θ_Ph,sat_ is phenol saturation coverage, Δ*G* (kJ/mol) is the adsorption free energy of phenol, *C*_Ph_ (M) is the bulk concentration of phenol in solution, and α is the Temkin parameter that accounts for lateral interactions between adsorbed phenol. It was shown with DFT that the gas-phase phenol adsorption energy on Pt(111) decreases linearly as a function of coverage due to lateral repulsion.^52^ The CVs with the increasing phenol concentration (Figures S15 and S16) and additional experiment and fit details are provided in the Supporting Information. The isotherm shapes in Figure 2 slightly contrast those found in the aforementioned study.^26^ Here, a single saturation coverage is reached at phenol concentrations of ∼0.1 mM and higher. This discrepancy could be due to differences in the electrochemical cell setups, electrolyte compositions, and catalyst form-factors used. The Δ*G*_θ=0_, α, θ_sat_, and Δ*G*_sat_ values for phenol on Rh and Pt, determined from fitting of the phenol adsorption isotherms in Figure 2 with eq 1, are summarized in Table 2. While the Δ*G*_θ=0_ for phenol becomes more positive (weaker) with increasing pH, the Δ*G*_θ=sat_, which accounts for the α parameter at saturated phenol coverages, becomes slightly more negative (stronger). The α parameter that gives the best fit also decreases with pH, starting at 12 and 10 at pH 5 for Pt and Rh, respectively, and becoming zero for both at pH 13. The decreasing α parameter suggests that there is a pH dependence on the adsorption configuration of phenol on both catalysts, which is consistent with the idea that the pH-dependent dissociation of phenol to phenoxide and increasing electric field strength with pH could induce such changes. The Δ*G*_θ=sat_ for phenol is also slightly stronger on Rh than Pt at each pH and exhibits a higher average nominal phenol saturation coverage of θ_sat_ = ∼0.81 on Rh compared to θ_sat_ = ∼0.70 on Pt at each pH. We note that coverage measurements, using loss of H_UPD_ area, is recorded at potentials higher than those at which ECH electrolysis is performed. However, it is reasonable to suggest that the comparative coverage trends between Pt and Rh as a function of pH would remain valid. Several studies have attributed the enhanced phenol ECH activity on metal alloy and metal/metal-oxide catalysts in comparison to a single metal catalyst to a composition-dependent increase in phenol coverage.^15,17,20,21,29^ The slightly higher phenol coverage and adsorption strength on Rh as compared to Pt at alkaline pH may therefore contribute, in part, to the observed higher phenol ECH activity. Another potential explanation for the enhanced activity on rhodium in the alkaline electrolyte is that phenol ECH does not proceed through the LH mechanism on Rh at these conditions, as is proposed for Pt.^12−14^

**Table 2:** Δ*G*_θ=0_, α, θ_sat_, and Δ*G*_θ=sat_ Values Obtained from Fit of Phenol Adsorption Isotherms with eq 1

	Δ*G*_θ=0_ phenol (kJ/mol)	α	θ_sat_	Δ*G*_θ=sat_ phenol (kJ/mol)
Platinum				
pH 5	–33	12	∼0.7	–24.6
pH 9	–34	6	∼0.73	–29.62
pH 13	–27	0	∼0.66	–27.0
Rhodium				
pH 5	–37	10	∼0.85	–28.5
pH 9	–35	4	∼0.8	–31.8
pH 13	–31	0	∼0.77	–31.0

If we consider the general hydrogenation of an adsorbed reactant, transfer of an H to that adsorbed species can occur through a direct addition of an adsorbed H, which is a sequential proton/electron transfer, through the LH mechanism.^13,14^ The H addition to an adsorbed species can also be accomplished through a concerted proton/electron transfer, where the H is abstracted from a solution-phase species. This is analogous to the Eley–Rideal (ER) mechanism for heterogeneous reactions.^53^ Phenol ECH on both Pt and Rh could potentially proceed through either an LH- or ER-type mechanism. There is a tendency in the phenol ECH literature to analyze the mechanistic progression of the reaction only from the viewpoint of an LH-type mechanism.^12−14^ This is likely due to continual comparisons to TCH reactions. It is possible, and even likely, that a change in electrolyte pH, from one end of the scale to the other, could induce a change in the dominant mechanism away from the assumed HAT-mediated LH-type mechanism. As electrolyte pH is increased, the barrier for the formation of H_ads_ increases due to the change in the source of that H, from H_3_O^+^ to H_2_O. This will result in slower H-adsorption kinetics at higher pH.^33,37,38^ It is likely, even at pH 1, that phenol ECH, if occurring only through a surface-limited HAT mechanism, is already limited by H-coverage due to its competitive adsorption with strong binding phenol, as evidenced by the high phenol coverages, as shown in Figure 2. It would be expected that the H-phenol competitive adsorption would also be affected by the pH-dependent relative concentrations of phenol/phenoxide in solution at electrolyte pH around phenol’s p*K*_a_. Combining the slower rate of H-adsorption, competitive adsorption between H and phenol, and the increasing concentration of negatively charged phenoxide, it makes sense, then, that ECH rates significantly decrease with increasing pH on Pt. Despite having a slightly stronger HBE, it has been shown that the H adsorption kinetics are slower on Rh than on Pt in both acid and base.^54,55^ This could possibly be a consequence of the nearly overlapping H_UPD_ and oxidation potentials on Rh and/or the stronger binding of water in the double layer on Rh than Pt.^26,55^ This could partially explain the lower ECH activity on Rh at low pH compared to Pt. However, if we were to continue with the logic derived from analyzing reaction kinetics from the viewpoint of an LH-type ECH mechanism, we would expect that the drop in ECH activity on Rh with increasing pH would be even more significant than that observed for Pt. As we observe the opposite response, seen in Figure 1B, we hypothesize that for Rh, there is a shift in the dominant mechanism by which H is transferred to the adsorbed phenol when moving to alkaline pH electrolytes. Specifically, the proton/electron transfer to adsorbed phenol is driven by a concerted transfer from a solution phase species in an ER-type mechanism. Support for this hypothesis is provided by a careful analysis of the observed FE and ECH PCD trends with pH on Rh, Figure 1B. The peak performance occurs at a pH just below the p*K*_a_ of phenol, 10.0. The conversion rates at pH 10 and above (Figure S12), where phenoxide is the dominant species, remain higher than or within standard deviation of the rates in acid, where phenol is the dominant species. This suggests that the peak at pH 9 and is not due to greater inactivity of phenoxide compared to phenol. When the electrolyte is at the p*K*_a_ of a dissociating functional group of a molecule, the reversibility for dissociation/reassociation of that functional group is the highest.^56^ Therefore, as the pH of the electrolyte approaches the p*K*_a_ of phenol, the barrier for near-surface phenol to act as a regenerative proton shuttle between water and adsorbed phenol will be minimized. In this process, the dissociating group on phenol acts as a catalyst for the abstraction of H from water or a buffer species. It is by this process that an ER-type ECH mechanism is promoted. At alkaline pH, where the barrier for H_ads_ formation is high and the competitive adsorption between H and phenol is shifted, it is likely that the barrier for ECH is lower for an ER-type hydrogenation than a LH-type mechanism.

**Figure 3:**
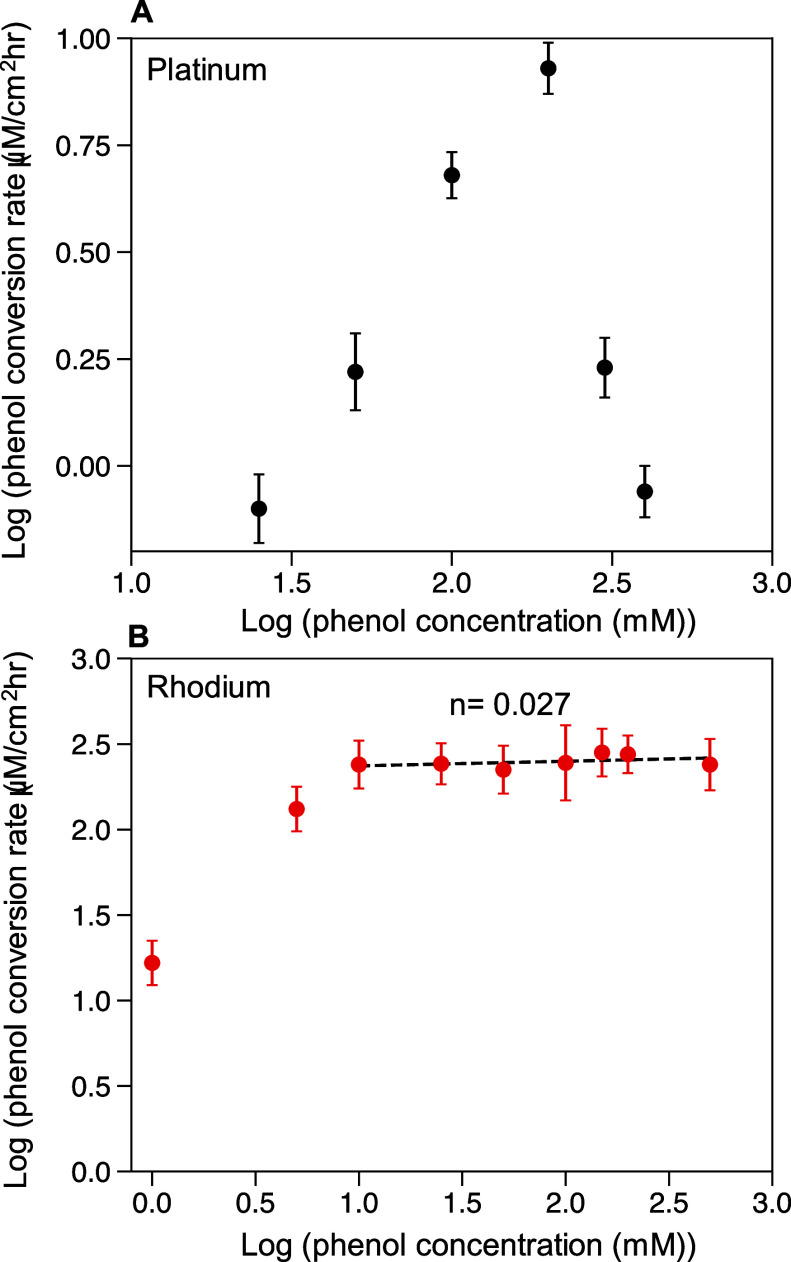
Phenol conversion rate vs phenol concentration for phenol ECH in pH 9 (0.6 M borate buffer), from electrolysis performed at −0.1 V vs RHE for 1 h with varying concentrations of phenol in the catholyte, on (A) platinum wire and (B) rhodium wire. Product concentration was too low for detection on the platinum wire with phenol concentrations <25 mM. Conversion was kept below 10% for all runs. The error bars represent standard deviation of at least 3 replicates.

Further support for a difference in the ECH mechanism at high pH for Pt and Rh is shown in Figure 3. Here, the reaction order is extracted from a plot of phenol conversion rate versus phenol concentration. From the isotherms in Figure 2, coverage of adsorbed phenol roughly scales with the phenol solution concentration. For an LH-type mechanism, phenol ECH rates should exhibit a positive reaction order at low phenol coverages and a negative reaction order at high phenol coverages.^14,57^ This is due to the phenol-coverage-dependent availability of H_ads_ for the HAT-mediated hydrogenation of adsorbed phenol. An ER-type mechanism will exhibit a positive reaction order at low phenol coverages and a zero reaction order at higher coverages.^17,57^ Derivations of the LH and ER rate expressions that describe the reaction order can be found in the Supporting Information. These rate expressions are used to qualitatively guide our interpretation of the phenol ECH mechanism from the plots of phenol ECH conversion rate versus phenol concentration in Figure 3. Figure 3A shows that on Pt at pH 9, the phenol ECH conversion rate is the positive order in the phenol concentration between 25 and 200 mM, and then becomes the negative order at phenol concentrations greater than 200 mM, consistent with a LH-type mechanism. Figure 3B shows that on Rh at pH 9, the phenol ECH conversion rate is positive order at phenol concentrations between 1 and 10 mM, and then becomes zero order (0.027 from the empirical power law) from 10 to 400 mM, which is consistent with an ER-type mechanism. Phenol concentrations above 400 mM degraded the anion exchange membrane. This result strongly supports our hypothesis of a pH-dependent shift in the dominant ECH mechanism for Rh.

The underlying source of this mechanism shift observed on Rh but not on Pt is not yet fully understood. The lower phenol coverage on Pt (Table 2), and the lower barrier for Tafel recombination than ECH on Pt at alkaline pH, evidenced by the decreasing FE for ECH with increasing pH (Figure 1), are potential contributing factors to why a switch from LH to ER mechanism is not observed on Pt. Further investigation, including detailed computational analysis, is needed to determine the true source of this switch.

We wish to briefly discuss the role of buffering components in the electrolyte for phenol ECH. Specifically, their role in maintaining electrolyte pH over long electrolysis periods and their impact on ECH and competing HER rates. The direct impact of buffers on phenol ECH rates remains relatively unknown. They are generally considered to be innocent spectator components whose only role is to maintain the electrolyte pH at a constant value. In intermediate pH electrolytes, a buffered solution whose pH is close to the buffer p*K*_a_ has been shown to promote HER compared to operating at a pH far from the p*K*_a_ of the buffer.^58^ This is because the interfacial buffer ions can supply protons to the catalyst surface, mitigating the overpotential that results from a local pH gradient.^58^ When operating at the buffer’s p*K*_a_, the Surendranath group found that buffers like phosphate outcompete H_2_O and H_3_O^+^ as proton donors toward HER on Au and Pt, enabling HER rates in neutral phosphate buffers close to those in 0.1 M HClO_4_.^39^ It is possible, then, that the buffer species may actively participate in phenol ECH, either by modulating the HER rates or by acting as a proton source for ECH. A previous report of phenol ECH on Rh at pH 10 was performed in acetate buffer adjusted with KOH.^12^ At pH 10, however, acetate is far from its p*K*_a_ value of 4.76 and has little buffering capacity. In this case, the acetate was simply affecting the ionic strength of the electrolyte and was not acting as a buffer or behaving as a proton shuttle.

**Figure 4:**
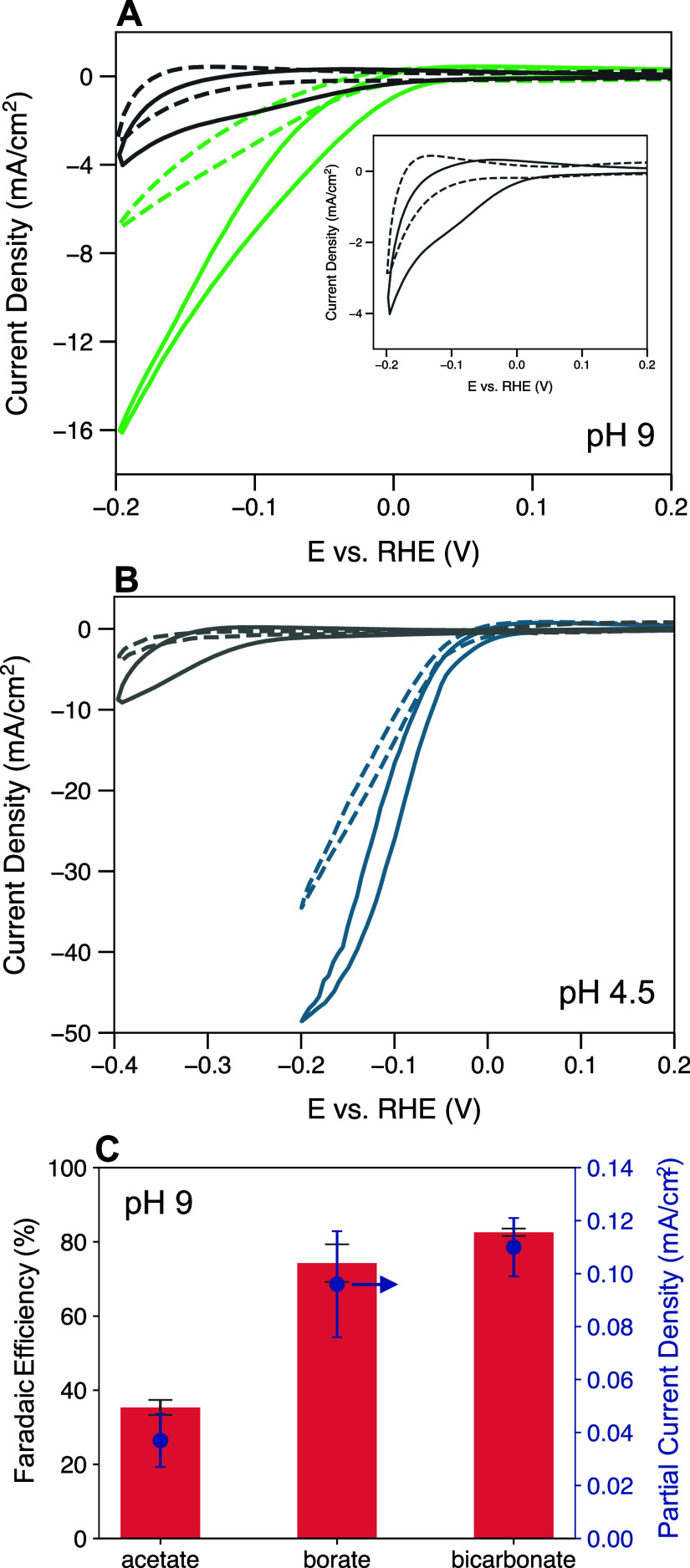
Polarization curves on Rh wire with 15 mM phenol (solid lines) and without phenol (dashed lines) in (A) pH 9 0.6 M acetate buffer (gray) and pH 9 0.6 M borate buffer (green) [inset: zoom-in on pH 9 0.6 M acetate buffer with (solid) and without (dashed) phenol] and (B) pH 4.5 0.1 M HClO_4_/KClO_4_ (gray) and pH 4.5 0.6 M acetate buffer (blue), scan rate = 50 mV/s (C) FE and PCD of phenol ECH on Rh/RVC WE in each of pH 9 acetate, borate, and bicarbonate after electrolysis at −0.2 V vs RHE for 1 h with 15 mM phenol in the catholyte. The error bars represent standard deviation of at least 3 replicates.

In Figure 4, we begin to analyze the possible impact of noninnocent buffer behavior for phenol ECH. The dashed lines in Figure 4A show HER polarization on Rh at pH 9 using acetic acid/acetate buffer and boric acid/borate buffer without any phenol present in solution. At pH 9, the boric acid buffer is near its p*K*_a_ of 9.24 and therefore has a high buffering capacity, while the acetic acid is far past its p*K*_a_ of 4.76, and we therefore consider it nonbuffered. The buffering capacity of acetic acid/acetate buffer as a function of pH is detailed in the Supporting Information. The HER activity, Figure 4A, is higher in the boric acid/borate buffer (dashed green line) than the acetic acid/acetate buffer (dashed black line). This demonstrates the noninnocent behavior of buffers, acting as a low barrier proton donor when the electrolyte pH is near the p*K*_a_ of the buffering species.

If phenol is added to the electrolytes with the inactive buffer (pH 9 acetate buffer solution; solid gray line in Figure 4A), the current density slightly increases. This increase is likely due to phenol ECH, indicated by the presence of the cathodic shoulder that is uncharacteristic of the HER (see inset of Figure 4A). When phenol is added to the pH 9 boric acid/borate buffer solution (solid green line), the current density increases more significantly than the nonbuffered case. This increased current must be due either to phenol ECH or increased HER activity in the presence of phenol. The solid green polarization curve in Figure 3A is performed at the same conditions as the phenol ECH electrolysis in Figure 1, where the FE and PCD are ∼74% and 0.096 mA/cm^2^ in the pH 9 boric acid/borate buffer. However, in the pH 9 acetic acid/acetate buffer, the FE and PCD are found to be ∼35% and 0.037 mA/cm^2^. The pH change of the pH 9 borate-buffered electrolyte was +0.2 pH units over the course of the 1 h electrolysis. The pH change of the pH 9 acetate buffer electrolyte was +0.6 pH units, ruling out a significant increase in bulk pH as a cause for the lower activity. However, one of the effects of the buffer capacity of the added buffer could be to impact the fluctuation of the interfacial pH during ECH electrolysis. In alkaline electrolytes, the dissociation of water to provide H for hydrogenation forms OH^–^ at the electrochemical interface, locally raising the pH. It is possible that in the acetate-containing electrolyte at alkaline pH, the passivity of the buffering species is not able to prevent an interfacial pH rise, leading to an ECH operational pH higher than the bulk. This would result in a lower ECH activity in comparison to an electrolyte containing an active buffer species that would effectively prevent this interfacial pH rise, i.e., borate/boric acid buffer.

The local pH during HER has been shown to increase to >12,^36,59^ a pH at which our results in Figure 1B, show that phenol ECH rates on Rh are diminished. In alkaline electrolytes around the p*K*_a_ of phenol, a pH increase at the interface could shift the dissociative equilibrium toward phenoxide, making the fraction of phenol dissociated at the interface different from the bulk. The FE of phenol ECH in bicarbonate buffer (p*K*_a_ 10.3) is also shown in Figure 4C as an additional example of phenol ECH in a buffer, whose p*K*_a_ is close to the electrolyte pH. The much higher FE in the borate and bicarbonate buffer at pH 9 compared to the acetate buffer at pH 9 shows that the increase in current density in the presence of phenol in the pH 9 borate buffer (Figure 4A) must be due predominantly to phenol ECH. If the increased current was due to HER, then a lower FE should have been observed. These results point to two possible roles of buffer species during phenol ECH: (1) proton shuttle and (2) maintaining a constant interfacial pH. The proximity of the electrolyte pH to the p*K*_a_ of the buffer species defines the degree to which the buffering component acts in these roles. If the electrolyte pH is near the p*K*_a_ of phenol, then phenol itself can also act in these roles.

**Figure 5:**
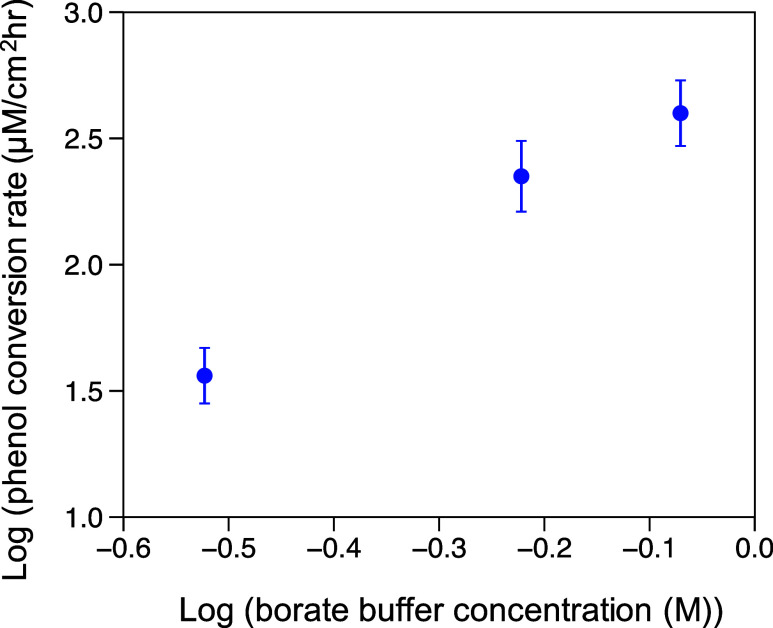
Phenol ECH conversion rate versus borate buffer concentration for ECH on rhodium at pH 9. Phenol ECH was performed on rhodium wire at −0.1 V vs RHE with 50 mM phenol in the catholyte, with borate buffer concentrations of 0.3, 0.6, and 0.85 M in the 0.1 M KClO_4_ supporting electrolyte. The error bars represent the standard deviation of at least 3 replicates.

**Scheme 1:**
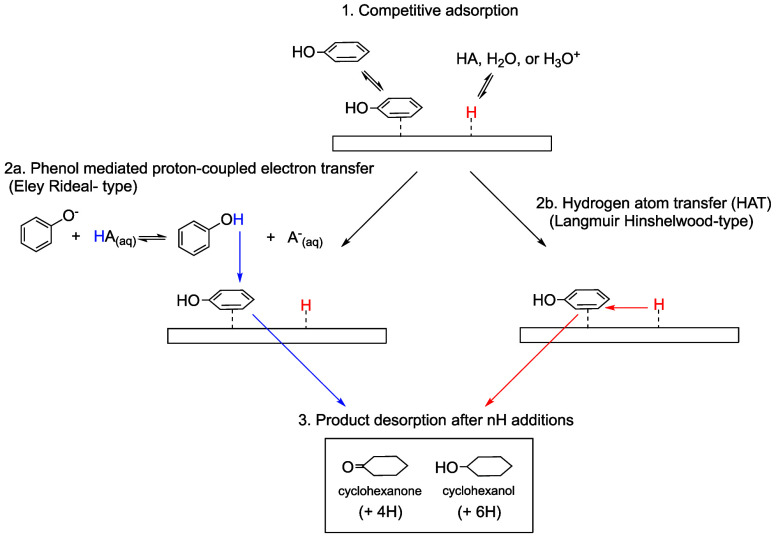
Proposed Mechanisms for Phenol ECH by (1) Competitive Adsorption of Phenol and H Followed by Either (2a) Phenol-Mediated PCET or (2b) HAT and (3) Desorption of Hydrogenation Products

The same phenol ECH enhancement in buffered versus nonbuffered electrolytes is observed when operating at pH 4.5. Figure 4B shows the polarization curves on Rh, with and without phenol, in both buffered (acetic acid/acetate) and nonbuffered (0.1 M HClO_4_/KClO_4_) electrolytes at pH 4.5. At pH 4.5, acetate buffer is near its p*K*_a_ value (4.76) and therefore has a high buffering capacity. We observe that in the absence of phenol, the acetate buffer has a significantly higher HER current density than the nonbuffered electrolyte, similar to the results observed for the boric acid/borate buffer in alkaline pH electrolytes. When phenol is added to the solution, there is a greater increase in current density in the buffered acetate electrolyte than the nonbuffered HClO_4_/KClO_4_ electrolyte. This increase could be due to phenol ECH, which occurs to an appreciable extent at pH 5 based on the results of Figure 1. The results of Figure 1 showed that the phenol ECH activity was higher at pH 9 than at pH 4.5, although both exhibit an increase in current density in the presence of phenol (Figure 4A,B). If operating through an ER mechanism, as evidenced in Figure 3, the ability of phenol to act as a proton shuttle to promote this pathway would be the greatest at its p*K*_a_ and may explain why the highest rate is observed around pH 9 and 10 for phenol ECH on Rh. Phenol has been previously shown to act as a proton shuttle toward benzaldehyde at the interface during benzaldehyde ECH and during Li-mediated ammonia synthesis.^60,61^ When the buffered electrolyte pH is near the p*K*_a_ of the buffer acid species for phenol ECH, the buffer acid is a possible H donor in the ER mechanism. From the ER rate expression (see the Supporting Information), the phenol ECH rate should have a positive order dependence in H^+^. In Figure 5, the phenol conversion rate shows a positive dependence in the borate buffer concentration, suggesting that the proton from the boric acid acts as a proton donor for phenol ECH and is also further evidence for an ER mechanism on Rh at pH 9. The increasing boric acid concentration also increases its capacity as a buffer, which could help to further mitigate the local pH change caused by H_2_O dissociation during HER, which would otherwise reduce phenol ECH rates as evidenced by the decreasing rates past pH 10 on Rh, as shown in Figure 1B. Scheme 1 illustrates the proposed mechanism for phenol ECH on Rh near pH 10 in which phenol mediates proton transfer from the buffer acid to surface adsorbed phenol in a PCET step (ER-type) (2a) and through a HAT step (LH- type) (2b).

Figure S18 shows polarization curves in buffered and unbuffered electrolytes on Pt wire both with and without phenol. Like Rh, we see the expected increase in HER current when using a buffered versus nonbuffered electrolyte in the absence of phenol. The current slightly increases when phenol is added to the nonbuffered electrolytes at both pH 4.45 and pH 9. The slight increase in current could be due to phenol ECH or from the ability of phenol to act as a buffer at the interface to improve HER. In the buffered electrolytes at both pH 4.45 and pH 9, where HER rates are much faster compared to the nonbuffered, the overall effect of adding phenol is a slight decrease in current density, likely because phenol ECH rates are comparatively lower.

The results of Figures 4 and 5 reveal the role of the buffer in defining, in part, the phenol ECH activity. The peak activity of phenol ECH on Rh at pH 9 is observed only in buffered electrolytes in which buffer acid p*K*_a_ ≈ pH. The phenol ECH activity further depends on the buffer concentration, likely due to its role in mitigating local pH fluctuations and acting as a H-donor in an ER-type mechanism (Scheme 1). Thus, the buffer identity and concentration are critical parameters that must be considered in the ECH system.

## Conclusions

In this work, we show that Pt and Rh ECH electrocatalysts exhibit contrasting pH trends for phenol ECH, with the highest phenol ECH activity on Pt observed in acidic electrolyte near pH 5, and the highest on Rh in the alkaline electrolyte at pH 9. The diminished activity on Pt in alkaline pH is attributed to the slower rate of H-adsorption in the base, competitive adsorption between phenol and H_ads_, and increased concentration of phenoxide at high pH. If following an LH mechanism, the same reasoning cannot explain the trend in pH on Rh, as H-adsorption rates were previously found to be even slower on Rh than Pt in base.^55^ Phenol adsorption isotherms also suggest only small differences in the phenol adsorption coverage and strength between the two metals. Kinetic analysis supports a LH mechanism for phenol ECH on Pt. In contrast, our kinetic analysis supports an ER-type mechanism on Rh at pH 9, in which adsorbed phenol is hydrogenated by a solution-phase proton donor, based on the observed zero order dependence in phenol at high phenol coverages. The observed positive order dependence in H^+^ (boric acid) on Rh at pH 9 is likely due to the buffer’s role in regulating interfacial pH and acting as a potential proton donor. We hypothesize that the peak activity on Rh is observed near pH 9 because of its proximity to phenol’s p*K*_a_, at which near-surface phenol can act as a proton shuttle from the buffer to adsorbed phenol. This work highlights the need for standardizations of the buffer and electrolyte composition for ECH reactions in order to compare activity across research groups. Furthermore, an LH mechanism should not be immediately assumed for non-Pt-containing or mixed metal catalysts, which are becoming increasingly used in the literature. Based on the presented results, a strategy for enhancing the rates and selectivity of hydrocarbon ECH is by tailoring both the electrolyte and catalyst to target specific functional groups on the reactant molecules, driving targeted ECH through PCET-type H-transfers under conditions where HER is minimized.

## Supporting Information Available

Experimental details including picture of custom H-Cell, buffer preparation, electrode characterization, additional CVs, additional data corresponding to the electrolysis experiments of Figure 1, and kinetic models (PDF)
