# Supplementary material for: pH-Mediated Solution-Phase Proton Transfer Drives Enhanced Electrochemical Hydrogenation of Phenol in Alkaline Electrolyte
Source: ACS Catal. 2024 Nov 1;14(22):16936–46. doi: 10.1021/acscatal.4c04874 (PMC11574755; doi:10.1021/acscatal.4c04874)
Supplement: Supplementary file 1 — cs4c04874_si_001.pdf [file cs4c04874_si_001.pdf]

Supporting Information:

pH-Mediated Solution-Phase Proton Transfer Drives Enhanced Electrochemical Hydrogenation of Phenol in Alkaline Electrolyte

Brianna Markunas[1], Taber Yim[1], Joshua Snyder[1,*]

[1]Chemical and Biological Engineering, Drexel University, Philadelphia, PA, 19104

*jds43@drexel.edu

Table of Contents:

S1: Buffer preparation and buffer capacity…………………………..…………………………...3
S2: Calculating electrochemically active surface area……………………………………………7
S3: Calculating faradaic efficiency and phenol conversion rate………………..…………………12
S4: Phenol adsorption isotherms…………………………………………………………………14
S5: Langmuir Hinshelwood and Eley-Rideal -type mechanisms and kinetic rate expressions for phenol ECH……………………..……………………………………………………………...17
S6: References……………………………………………………………………………………19

List of Figures

Figure S1: H-Cell set-up for electrolysis………………………………………………………..2
Figure S2: Theoretical titration curve for boric acid with KOH…………………………………..4
Figure S3: Acetate buffer capacity…………………………………………………………...5
Figure S4: Picture of RVC electrodes before and after rhodium deposition………………………6
Figure S5: CV of Pt/RVC electrode in 0.1 M $HClO_4$……………………………………………7
Figure S6: Scanning electron microscope image of Pt/RVC electrode, overlaid with colorized elemental mapping using energy dispersive spectroscopy…………………………………………8
Figure S7: Scanning electron microscope image of Rh/RVC electrode, overlaid with colorized elemental mapping using energy dispersive spectroscopy…………………………………………8
Figure S8: Scanning electron microscope image of Pt/RVC electrode……………………………9
Figure S9: Scanning electron microscope image of Rh/RVC electrode……………………………9
Figure S10: CVs on platinum wire with and w/out phenol at varying pH………………………10
Figure S11: CVs on rhodium wire with and w/out phenol at varying pH…………………………11
Figure S12: Phenol conversion rates on Pt/RVC and Rh/RVC…………………………………..12
Figure S13: Carbon balance for phenol ECH electrolysis……………………………………...13
Figure S14: Total current density from phenol ECH on Pt/RVC and Rh/RVC…………………13
Figure S15: CVs on platinum wire at varying phenol concentrations……………………………14
Figure S16: CVs on rhodium wire at varying phenol concentrations……………………………15
Figure S17: Plots of Q_{ads} and Q_{des} on platinum and rhodium ………………..……………………..15
Figure S18: Polarization curves on platinum wire at pH 4.5 and pH 9, with and w/out phenol…16

List of Tables

Table S1: Average ECSA of Pt/RVC and Rh/RVC electrodes……………………………………7

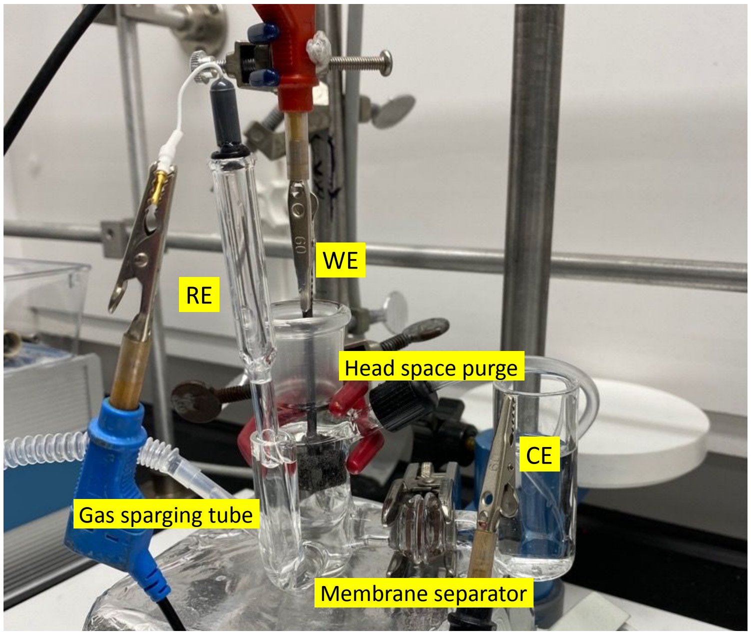

Figure S1. Electrochemical H-cell set-up for electrolysis

S1. Buffer preparation and buffer capacity

The theoretical titration curves were calculated following methodology from Reference 1 Ch.10[1]. For the dissociation of a monoprotic weak acid, [HA] with an acid dissociation constant K_a,

$$HA \rightleftharpoons H^+ + A^- \quad (K_a)$$
$$K_a = \frac{[H^+][A^-]}{[HA]}$$

Writing a mass balance on the formal concentration of [HA] yields:

$$F_{HA} = [HA] + [A^-]$$

The fraction of dissociation of HA and A- can be written as

$$\alpha_{HA} = \frac{[HA]}{[HA] + [A^-]} = \frac{[H^+]}{[H^+] + K_a}$$
$$\alpha_{A-} = \frac{[A^-]}{[HA] + [A^-]} = \frac{K_a}{[H^+] + K_a}$$

For the titration of HA of volume Va and concentration C_a, with a strong base (KOH in this work) of volume V_b and concentration C_b, the charge balance is:

$$[H^+] + [K^+] = [A^-] + [OH^-]$$

The concentration of K^+ is then:

$$[K^+] = \frac{C_b V_b}{V_a + V_b}$$

After dilution with $C_b V_b$ moles of KOH to a total volume of $V_a + V_b$.
Similarly, the formal concentration of HA is

$$F_{HA} = [HA] + [A^-] = \frac{C_a V_a}{V_a + V_b}$$

Because $C_a V_a$ moles of HA are diluted to a total volume of $V_a + V_b$.
The concentration of A^- can be written in terms of its fractional composition:

$$[A^-] = \alpha_{A-} \cdot F_{HA} = \frac{\alpha_{A-} \cdot C_a V_a}{V_a + V_b}$$

Finally, substituting these expressions for $[K^+]$ and $[A^-]$ in the charge balance and rearranging gives:

$$\phi \equiv \frac{C_b V_b}{C_a V_a} = \frac{\alpha_{A-} - \frac{[H^+] - [OH^-]}{C_a}}{1 + \frac{[H^+] - [OH^-]}{C_b}}$$

Where φ is the fraction of titration that has been completed. We now have an equation that relates the volume of the titrant (KOH) to the concentration of H^+ in solution (pH). Similar calculations are derived for di- and tri-protic acids titrated with strong base. An example titration curve for boric acid used in this work is shown in Figure S2.

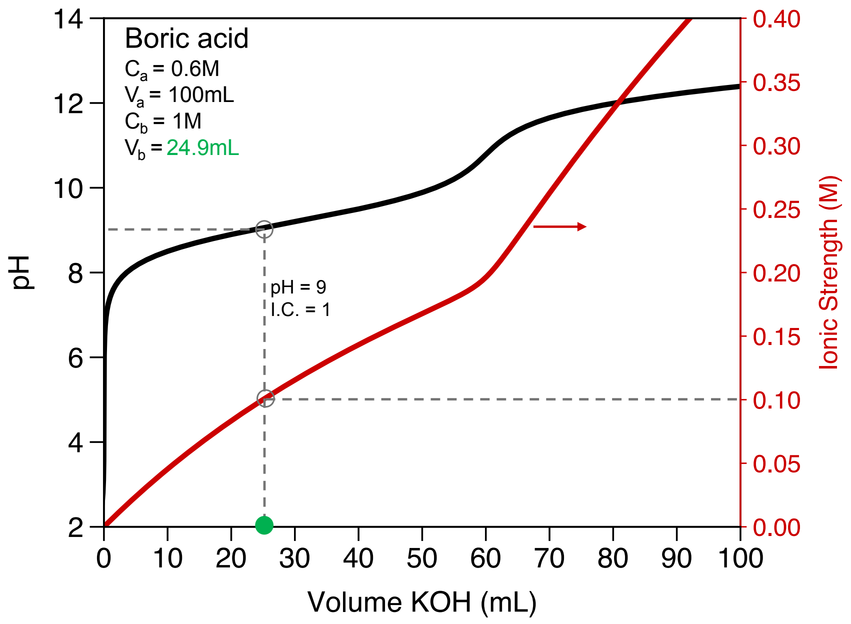

Figure S2. Theoretical titration curve for boric acid with KOH.

The capacity of a buffer can be defined as:

$$\text{Buffer capacity} = \frac{dn_b}{dpH} = -\frac{dn_a}{dpH} \quad 1$$

Where Ca and C_b are the number of moles of strong acid or strong base needed to change the pH by one unit.

$$\text{Buffer capacity} = 2.303 \times \left(\frac{K_W}{[H^+]} + [H^+] + \sum \frac{C_{buff} K_a [H^+]}{(K_a + [H^+])^2}\right) \qquad \text{Equation S1}$$

Where C_{buff} is the total concentration of [HA] + [A-]

Figure S3 shows the buffer capacity versus pH for acetic acid/acetate buffer. The buffering capacity is highest in the range of 3.8 to 5.8 and peaks when the pH = pKa (pKa = 4.76 for acetic acid). The buffering capacity also increases with increasing total concentration of the buffer species. In our work, the use of acetic acid/acetate buffer at pH 9, where it exists entirely as acetate, is purposely used as a non-buffered electrolyte in the polarization curves in Figure 4.

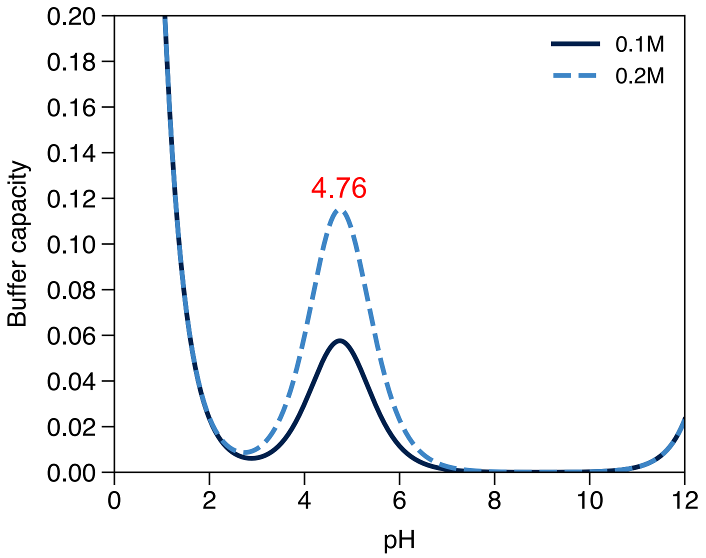

Figure S3. Acetate buffer capacity versus pH for 0.1 M (solid line) and 0.2M (dashed line) acetic acid/acetate buffer described by Equation S1

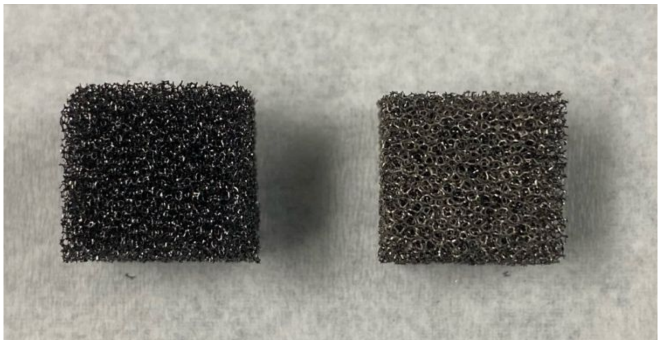

Figure S4. Bare RVC (left) and RVC after electrodeposition of rhodium (right)

S2. Calculating electrochemically active surface area

The electrochemically active surface area (ECSA) was calculated using the hydrogen underpotential deposition (H_{UPD}) method. The H_{UPD} charge associated with hydrogen desorption was integrated from the baseline corrected i vs t plot in the H_{UPD} potential window. The ECSA was determined by dividing the this charge by the specific charge of polycrystalline platinum (210 $\mu C/cm^2$) or rhodium (221 $\mu C/cm^2$)[2]. The method for electrodeposition of platinum or rhodium onto the RVC mesh resulted in electrodes with similar ECSA (see Table S1). Figure S5 shows a CV of a Pt/RVC electrode in 0.1 M $HClO_4$. The shaded area under the curve represents the H-desorption region (blue) and H-adsorption region (yellow) on platinum.

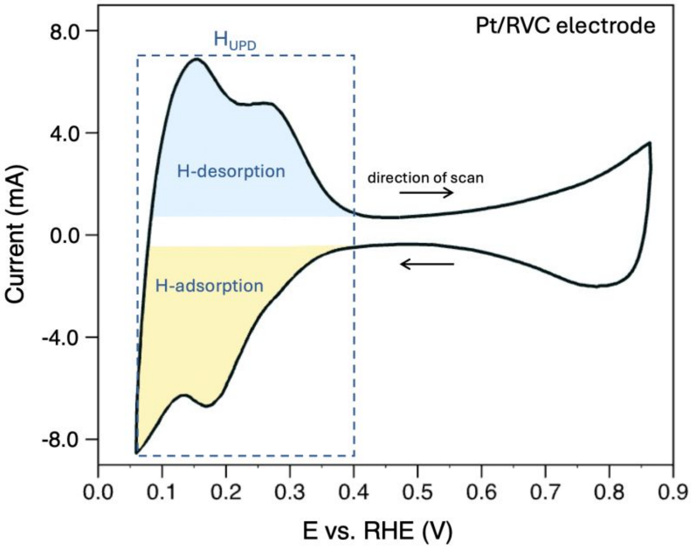

Figure S5: Cyclic voltammogram of a Pt/RVC electrode in 0.1 M $HClO_4$ from 0.05 V to 0.85 V vs RHE at a scan rate of 50 mV/s. The blue shaded region shows the current associated hydrogen desorption and the yellow shaded region shows the current associated with hydrogen desorption. The CV was taken in a single chamber FEP cell with a Ag/AgCl reference and Pt wire counter electrode, and purged with argon gas. 85% iR compensation was determined by EIS measurement and applied in the CV program.

Table S1: Average ECSA and standard deviation of 30 each of Rh/RVC and Pt/RVC electrodes

	Platinum	Rhodium
Average ECSA	87.43	88.91
Std Dev	4.12	7.65

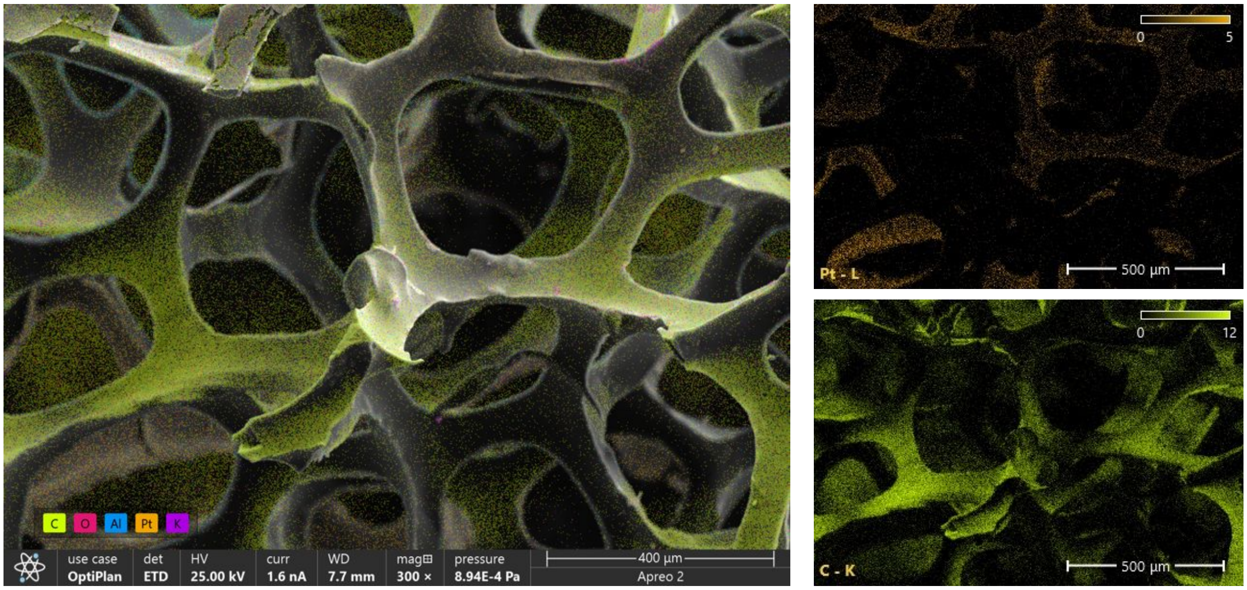

Figure S6. Scanning electron microscope image of platinum electrodeposited on RVC electrode, overlaid with colorized elemental mapping using energy dispersive spectroscopy. Green = carbon, orange = platinum.

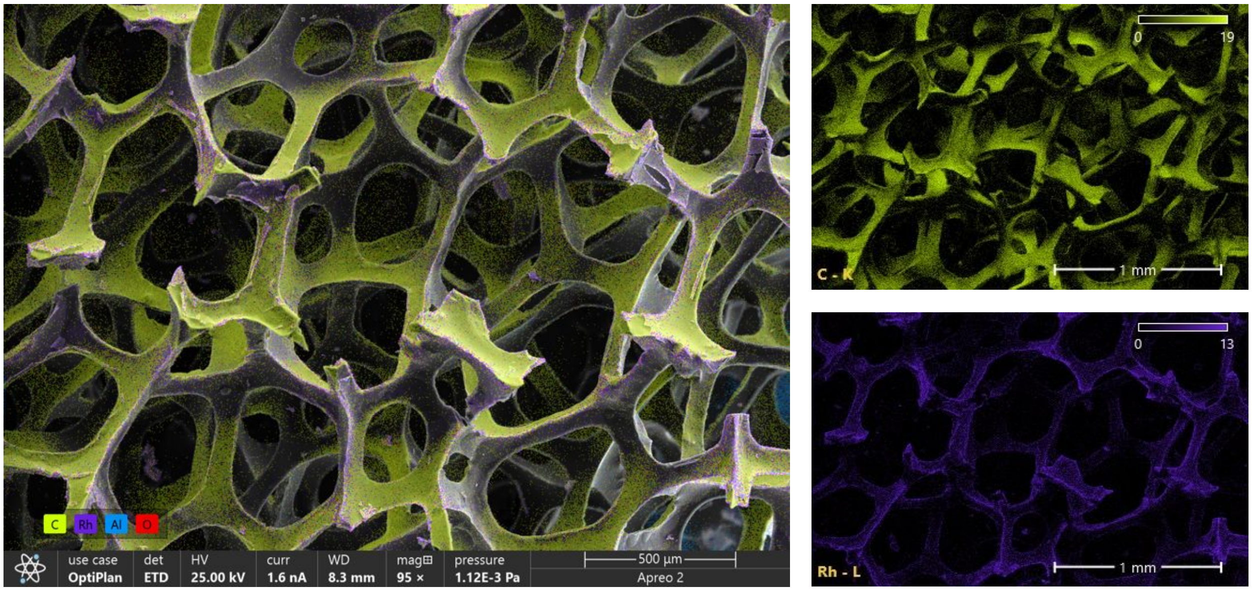

Figure S7. Scanning electron microscope image of rhodium electrodeposited on RVC electrode, overlaid with colorized elemental mapping using energy dispersive spectroscopy. Green = carbon. Purple = rhodium.

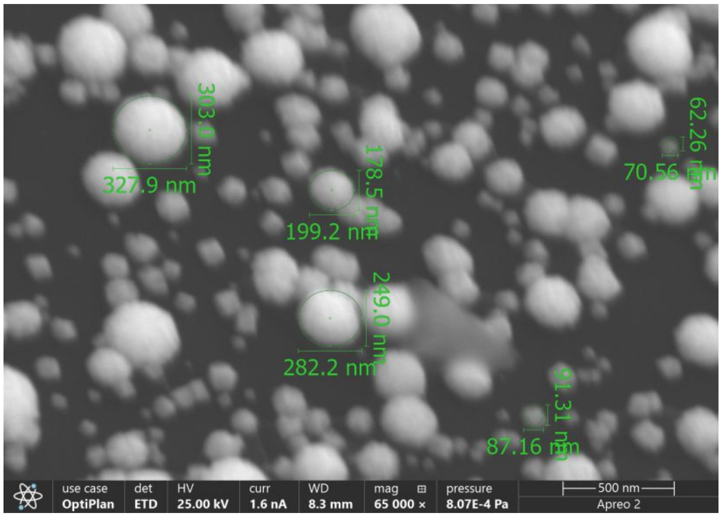

Figure S8: Scanning electron microscope image of platinum electrodeposited on RVC mesh. Platinum metal aggregates range from 31 nm to 164 nm.

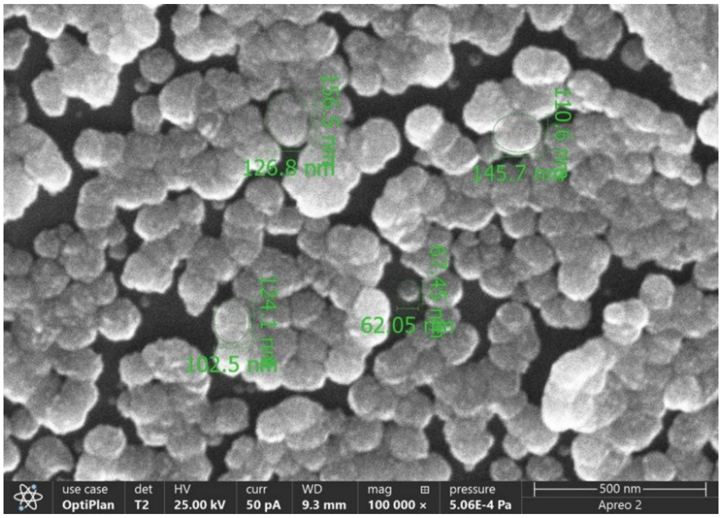

Figure S9: Scanning electron microscope image of rhodium electrodeposited on RVC electrode. Rhodium metal aggregates range from 31 nm to 78 nm.

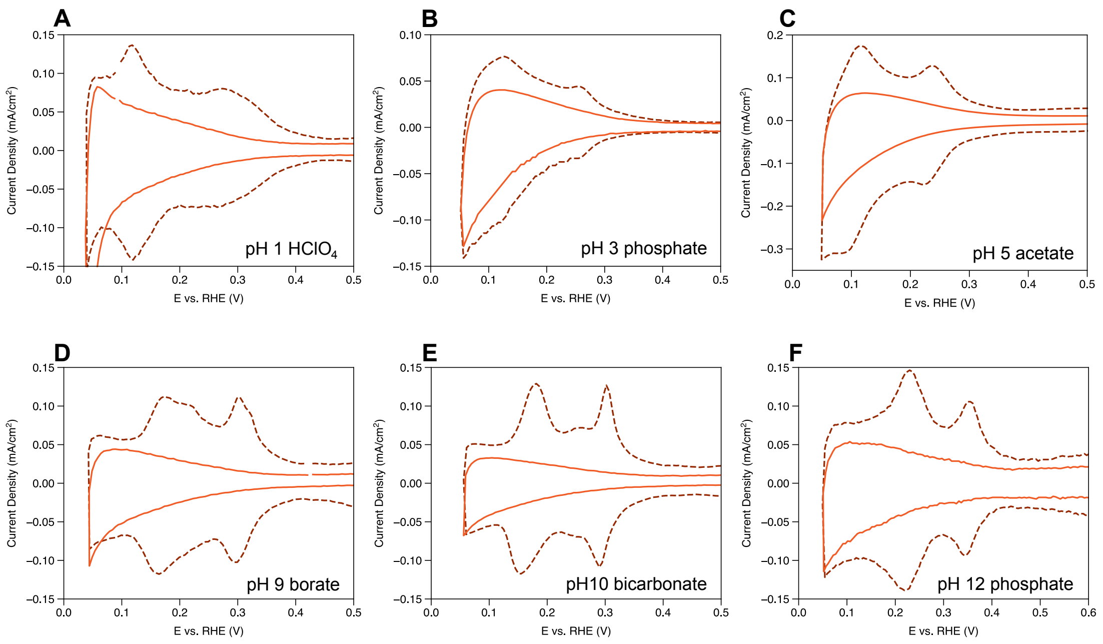

Figure 10: Cyclic voltammograms on platinum wire in buffered electrolyte with 15 mM phenol (solid) and without phenol (dashed). The polarization curves were taken in a single chamber FEP cell with a Ag/AgCl reference electrode and Pt wire counter electrode and purged with argon. The scan rate was 50 mV/s and an iR compensation of 85% was applied.

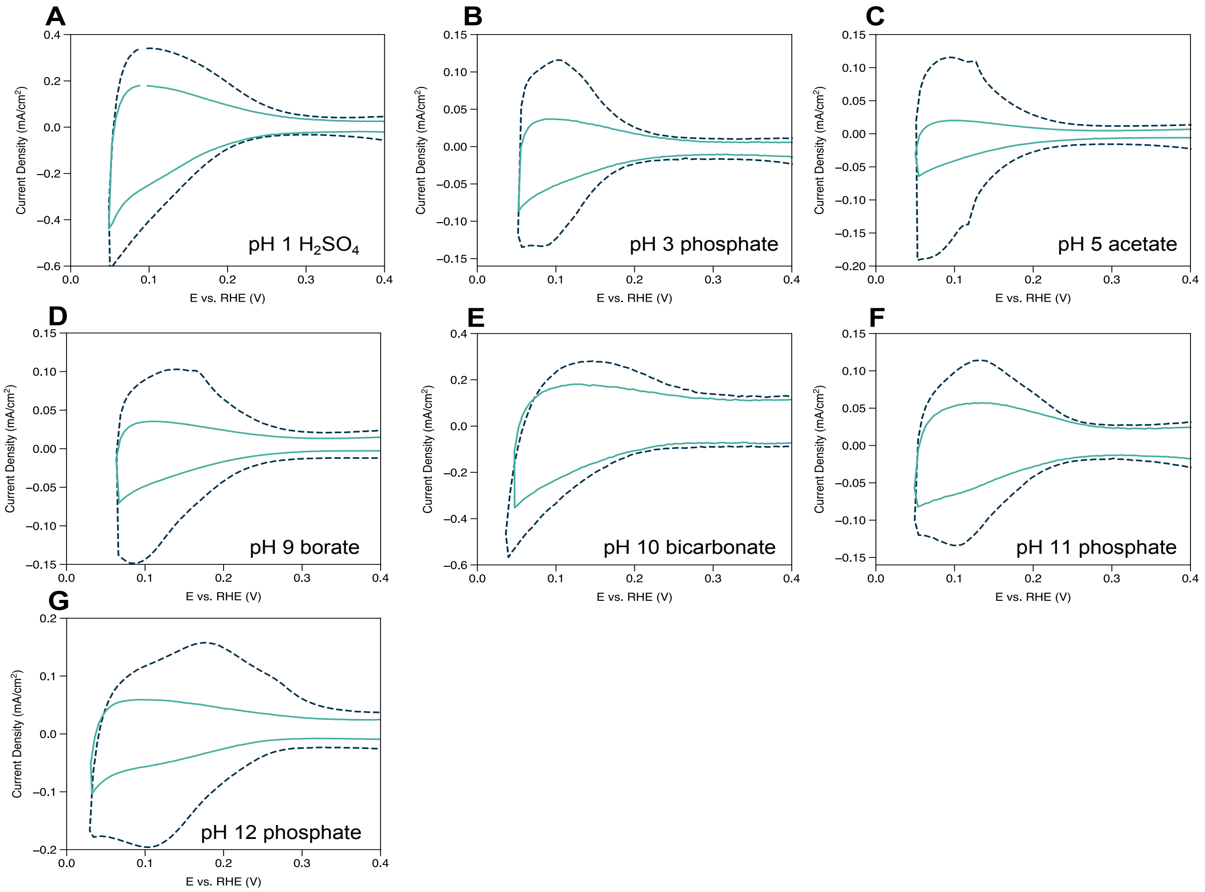

Figure S11: Cyclic voltammograms on rhodium wire in buffered electrolyte with 15 mM phenol (solid) and without phenol (dashed). The polarization curves were taken in a single chamber FEP cell with a Ag/AgCl reference electrode and Pt wire counter electrode and purged with argon. The scan rate was 50mV/s and an iR compensation of 85% was applied.

S3: Calculating faradaic efficiency and phenol conversion rate

$$\text{Faradaic efficiency (FE) (\%)} = \frac{\text{N x } n \text{ x F}}{\text{Q}} * 100$$

where N is the number of moles of product, *n* is the number of moles of electrons transferred per 1 mol of product formed (4 for cyclohexanone and 6 for cyclohexanol), F is Faraday's constant (96,485 C/mol), and Q (C) is the total charge passed during the electrolysis, integrated from the i vs. t plot.

$$\text{Phenol conversation rate} = \frac{\text{moles phenol converted to product}(\mu\text{M})}{\text{ECSA }(\text{cm}^2)*\text{time(s)}}$$

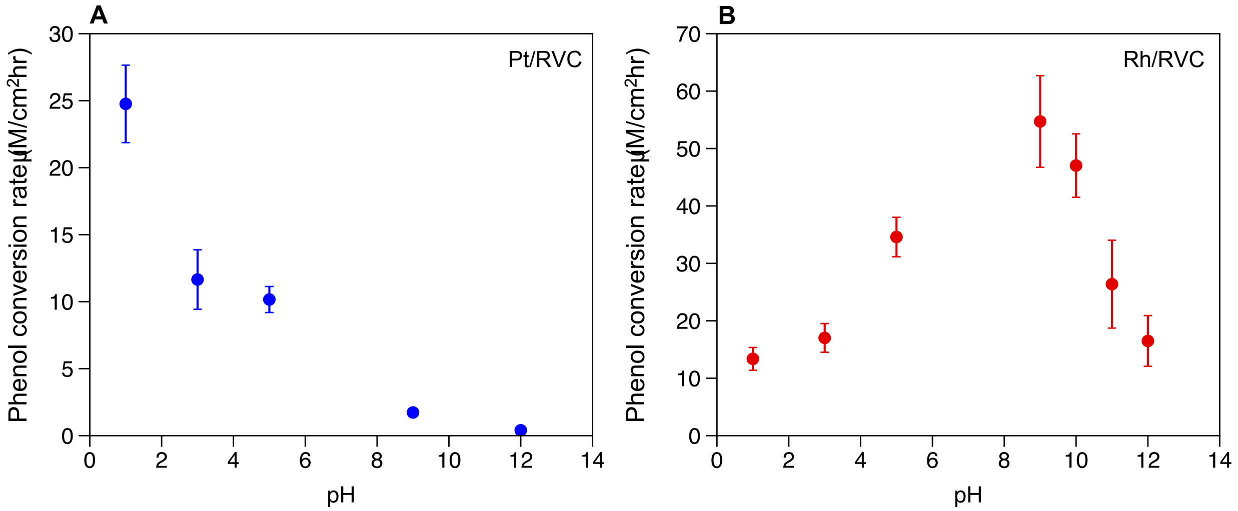

Figure S12: Phenol conversion rates at each pH, corresponding to the electrolysis experiments of Figure 1, in which phenol ECH was performed at -0.2 V vs RHE for 1 hour in a Nafion membrane-separated H-cell with 15 mM phenol in the catholyte at -0.2 V vs RHE for 1 hour on **(A)** Pt/RVC and **(B)** Rh/RVC. A Ag/AgCl reference electrode and Pt wire counter electrode were used and 85% iR compensation was applied. The error bars represent standard deviation of at least 3 repeats.

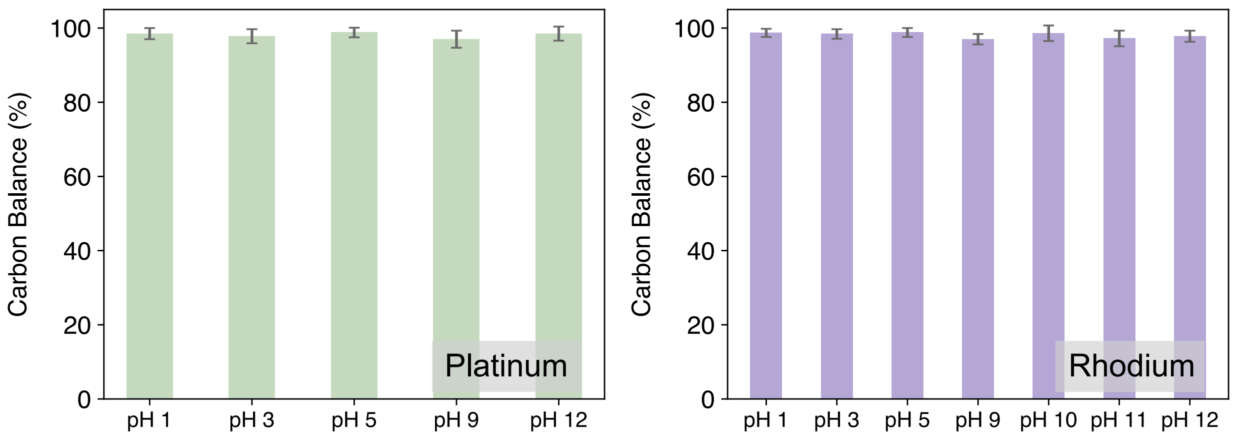

Figure S13: Average carbon balance (%) of three trials for electrolysis at each pH corresponding to experiments of Figure 1 for platinum (left, green) and rhodium (right, purple). The carbon balance ranges from 97-99 %, supporting completeness of our data in Figure 1 and the observance of no other hydrogenation products other than cyclohexanone and cyclohexanol. Minor losses may be from systematic/user error of the GC-MS analysis and quantification.

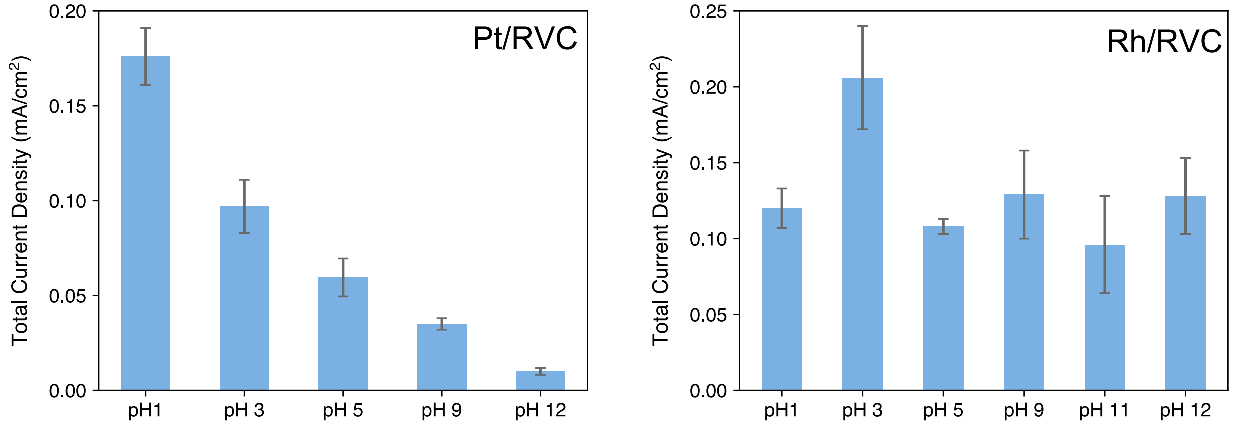

Figure S14: Total current density (combined HER and phenol ECH) for electrolysis on Pt/RVC (left) and Rh/RVC (right) corresponding to the electrolysis experiments in Figure 1, in which phenol ECH was performed at -0.2 V vs RHE for 1 hour in a membrane-separated H-cell with 15 mM phenol in the catholyte. A Ag/AgCl reference electrode and Pt wire counter electrode were used and 85% iR compensation was applied.

S4. Phenol adsorption isotherms

Figure S15 and Figure S16 show the cyclic voltammograms on a Pt wire and Rh wire, respectively, in pure electrolyte and with phenol concentrations varying 0 µM to 20 mM. The coverage of phenol on platinum and rhodium is assumed to be proportional to the fraction of the H_{UPD} charge that is inhibited in its presence, which is calculated by taking the difference between the H_{UPD} charge in pure electrolyte and the H_{UPD} charge in the presence of phenol. Several steps were taken to avoid adsorbing contaminants. The electrochemically cell parts were initially acid cleaned for 10 hours in concentrated nitric and sulfuric acid (1:1). The Pt and Rh wire electrodes were submersed in concentrated nitric acid followed by rinsing and sonication in DI water prior to each experiment. The Pt and Rh wires were also electrochemically cycled from 0.05 V to 1.1 V vs RHE at 250 mV/s in the pure electrolyte for at least 50 scans, and subsequent CVs were indicative of a clean electrode. The chemicals used, including phenol, buffer acids, and KOH, were newly acquired and of the highest available purity. The best fit in Figure 2 was obtained by minimizing the sum of the squared residuals.Figure S17 shows the HUPD charge obtained from taking either the charge from H adsorption (Q_{ads}) or H desorption (Q_{des}). They are nearly identical, indicating that no reaction between phenol and hydrogen is taking place in this potential range. In the case of rhodium, Q_{ads} is slightly higher than Q_{des} because the hydrogen adsorption region overlaps slightly with Rh_2O_3 reduction. Thus, Q_{des} charge is used in the isotherms. This is in agreement with the same analysis done by a previous group[3].

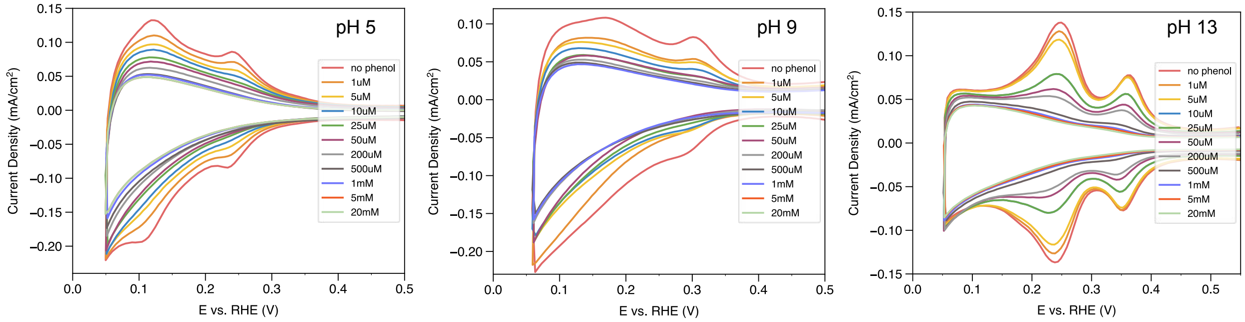

Figure S15: CVs on platinum wire with phenol concentration varying from 0 to 20mM, at pH 5 (0.6M acetic acid/ acetate), pH 9 (0.6 M boric acid/borate), and pH 13 (0.1 M KOH). The potential range is 0.05 V to 0.9 V vs RHE (cutoff at 0.5 V in the figure). The buffers were prepared according to the procedure detailed in S1. The polarization curves were taken in a single chamber FEP cell with a Ag/AgCl reference electrode and Pt wire counter electrode and purged with argon. The scan rate was 50 mV/s and an iR compensation of 85% was applied.

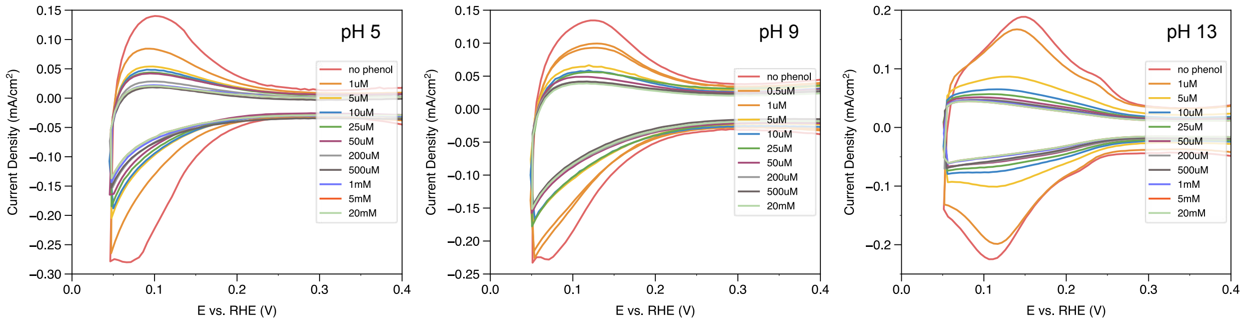

Figure S16: CVs on platinum wire with phenol concentration varying from 0 to 20 mM, at pH 5 (0.6 M acetic acid/ acetate), pH 9 (0.6 M boric acid/borate), and pH 13 (0.1 M KOH). The buffers were prepared according to the procedure detailed in S1. The potential range is 0.05 V to 0.9 V vs RHE (cutoff at 0.5 V in the figure). for The polarization curves were taken in a single chamber FEP cell with a Ag/AgCl reference electrode and Pt wire counter electrode and purged with argon. The scan rate was 50 mV/s and an iR compensation of 85% was applied.

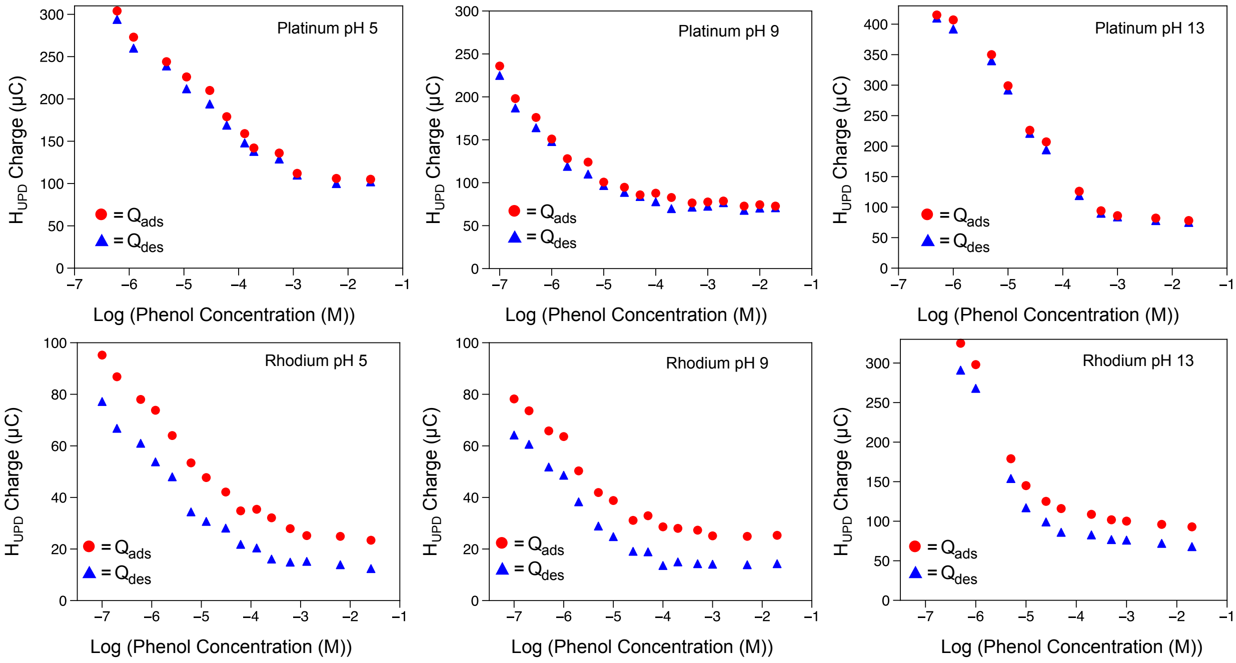

Figure S17: Plots for Q_{ads} (charge of H-adsorption) and Q_{des} (charge of H-desorption) on platinum (top) and rhodium (bottom) at pH 5, 9, and 13.

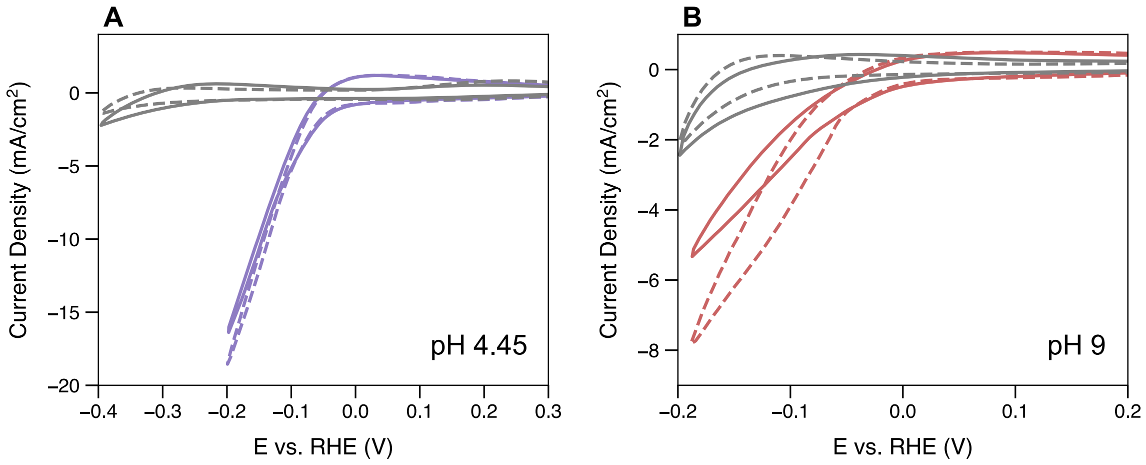

Figure S18: (**A**) Polarization curves on Pt wire in pH 4.45 unbuffered 0.1 M $HClO_4$/$KClO_4$ (grey) and pH 4.45 buffered 0.6 M acetate buffer (purple) electrolytes with and without phenol (dashed = no phenol, solid = 15 mM phenol) **(B)** Polarization curves on Pt wire in pH 9 acetate buffer (grey) and pH 9 borate buffer (red), with and without phenol (dashed = no phenol, solid = 15 mM phenol). The polarization curves were taken in a single chamber FEP cell with a Ag/AgCl reference electrode and Pt wire counter electrode and purged with argon. The scan rate was 50 mV/s and an iR compensation of 85% was applied.

S5. Langmuir Hinshelwood and Eley Rideal- type mechanisms and kinetic rate expressions for phenol ECH

Detailed below are the elementary steps for phenol ECH following a Langmuir Hinshelwood (LH) or Eley-Rideal (ER) (PCET-type). In both cases, the hydrogen evolution reaction (HER) is a competing reaction. We assume that the addition of the first H (either from surface adsorbed H in the LH mechanism or H^+ directly from a solution species in the ER mechanism) is the rate determining step.

Symbols:

= free site, Ph = phenol, PhH_1 = phenol with first H addition, PhH_n= phenol with n^{th} H addition. A "" directly following a species symbol represents an adsorbed species (i.e. Ph* = adsorbed phenol).

<u>Langmuir Hinshelwood mechanism:</u>

H adsorption: $H^+ + e^- + * \rightleftharpoons H*$ (K_1)

Phenol adsorption: $Ph + * \rightleftharpoons Ph*$ (K_2)

First H addition: $Ph* + H* + e^- \rightarrow PhH_1* + *$ (K_{LH}, RLS)

Product desorption: $PhH_n* \rightleftharpoons PhH_n + *$ (K_3)

H_2 evolution: $H* + H* \rightleftharpoons H_2 + 2*$ (K_4)

$$\theta_H = \frac{K_1 a_{H^+}}{1 + K_1 a_{H^+} + K_2 C_{Ph}}$$

$$\theta_{Ph} = \frac{K_2 C_{Ph}}{1 + K_1 a_{H^+} + K_2 C_{Ph}}$$

$$r_{LH} = k_{LH,f}\theta_{Ph}\theta_H$$

$$r_{LH} = \frac{k_3 K_1 a_{H^+} K_2 C_{Ph}}{(1 + K_1 a_{H^+} + K_2 C_{Ph})^2}$$

At low phenol coverages

$$r_{LH} = \frac{k_3 K_1 a_{H^+} K_2 C_{Ph}}{\left(1 + K_1 a_{H^+}\right)^2}$$; positive order in phenol

At high phenol coverages

$$r_{LH} = \frac{k_3 K_1 a_{H^+}}{K_2 C_{Ph}}$$; negative order in phenol

Eley-Rideal mechanism:

H adsorption: $H^+ + e^- + * \rightleftharpoons H*$ (K_1)

Phenol adsorption: $Ph + * \rightleftharpoons Ph*$ (K_2)

First H addition: $Ph* + H^+ + e^- \rightarrow PhH_1*$ $(K_{ER}$, RLS)

Product desorption: $PhH_n* \rightleftharpoons PhH_n + *$ (K_3)

H_2 evolution: $H* + H* \rightleftharpoons H_2 + 2*$ (K_4)

$$\theta_H = \frac{K_1 a_{H^+}}{1 + K_1 a_{H^+} + K_2 C_{Ph}}$$

$$\theta_{Ph} = \frac{K_2 C_{Ph}}{1 + K_1 a_{H^+} + K_2 C_{Ph}}$$

$$r_{ER} = k_{ER,f} \theta_{Ph} a_{H^+}$$

$$r_{ER} = \frac{k_3 a_{H^+} K_2 C_{Ph}}{1 + K_1 a_{H^+} + K_2 C_{Ph}}$$

At low phenol coverages

$$r_{ER} = \frac{k_3 K_2 a_{H^+} C_{Ph}}{1 + K_1 a_{H^+}}$$; positive order in phenol

At high phenol coverages

$$r_{ER} = k_3 a_{H^+}$$; zero order in phenol

S6. References

(1) Daniel C. Harris; Charles A. Lucy. Monoprotic Acid Base Equilibria. In *Quantitative Chemical Analysis*; W.H. Freeman and Company, New York, 2010; Vol. 58, p 177.

(2) Rand, D. A. J.; Woods, R. The Nature of Adsorbed Oxygen on Rhodium, Palladium and Gold Electrodes. *J. Electroanal. Chem. Interfacial Electrochem.* **1971**, *31* (1), 29–38. https://doi.org/10.1016/S0022-0728(71)80039-6.

(3) Akinola, J.; Barth, I.; Goldsmith, B. R.; Singh, N. Adsorption Energies of Oxygenated Aromatics and Organics on Rhodium and Platinum in Aqueous Phase. *ACS Catal.* **2020**, *10* (9), 4929–4941. https://doi.org/10.1021/acscatal.0c00803.
